# Empowering School Staff to Support Pupil Mental Health Through a Brief, Interactive Web-Based Training Program: Mixed Methods Study

**DOI:** 10.2196/46764

**Published:** 2024-04-23

**Authors:** Emma Soneson, Emma Howarth, Alison Weir, Peter B Jones, Mina Fazel

**Affiliations:** 1 Department of Psychiatry University of Oxford Oxford United Kingdom; 2 Department of Psychiatry University of Cambridge Cambridge United Kingdom; 3 School of Psychology University of East London London United Kingdom; 4 Faculty of Education University of Cambridge Cambridge United Kingdom; 5 Howard Community Academy Anglian Learning multi-academy trust Bury St Edmunds United Kingdom

**Keywords:** mental health, children, schools, teachers, training, digital intervention, pupil mental health, mental health training, intervention, empowerment, student, pupil, support, school staff, web-based training

## Abstract

**Background:**

Schools in the United Kingdom and elsewhere are expected to protect and promote pupil mental health. However, many school staff members do not feel confident in identifying and responding to pupil mental health difficulties and report wanting additional training in this area.

**Objective:**

We aimed to explore the feasibility of Kognito’s *At-Risk for Elementary School Educators*, a brief, interactive web-based training program that uses a simulation-based approach to improve school staff’s knowledge and skills in supporting pupil mental health.

**Methods:**

We conducted a mixed methods, nonrandomized feasibility study of *At-Risk for Elementary School Educators* in 6 UK primary schools. Our outcomes were (1) school staff’s self-efficacy and preparedness to identify and respond to pupil mental health difficulties, (2) school staff’s identification of mental health difficulties and increased risk of mental health difficulties, (3) mental health support for identified pupils (including conversations about concerns, documentation of concerns, in-class and in-school support, and referral and access to specialist mental health services), and (4) the acceptability and practicality of the training. We assessed these outcomes using a series of questionnaires completed at baseline (T1), 1 week after the training (T2), and 3 months after the training (T3), as well as semistructured qualitative interviews. Following guidance for feasibility studies, we assessed quantitative outcomes across time points by comparing medians and IQRs and analyzed qualitative data using reflexive thematic analysis.

**Results:**

A total of 108 teachers and teaching assistants (TAs) completed T1 questionnaires, 89 (82.4%) completed T2 questionnaires, and 70 (64.8%) completed T3 questionnaires; 54 (50%) completed all 3. Eight school staff members, including teachers, TAs, mental health leads, and senior leaders, participated in the interviews. School staff reported greater confidence and preparedness in identifying and responding to mental health difficulties after completing the training. The proportion of pupils whom they identified as having mental health difficulties or increased risk declined slightly over time (median_T1_=10%; median_T2_=10%; median_T3_=7.4%), but findings suggested a slight increase in accuracy compared with a validated screening measure (the Strengths and Difficulties Questionnaire). In-school mental health support outcomes for identified pupils improved after the training, with increases in formal documentation and communication of concerns as well as provision of in-class and in-school support. Referrals and access to external mental health services remained constant. The qualitative findings indicated that school staff perceived the training as useful, practical, and acceptable.

**Conclusions:**

The findings suggest that brief, interactive web-based training programs such as *At-Risk for Elementary School Educators* are a feasible means to improve the identification of and response to mental health difficulties in UK primary schools. Such training may help address the high prevalence of mental health difficulties in this age group by helping facilitate access to care and support.

## Introduction

### Background

In recent years, there has been an increased emphasis on the role of schools in supporting children’s mental health [1-3]. This enhanced focus has been driven in large part by an apparent increase in mental health difficulties (including behavioral, social, and emotional difficulties) present in school-aged populations [4-6]—a concern that became increasingly prominent in the context of the COVID-19 pandemic and the associated school closures and social distancing measures [7,8]. There is also a growing recognition of the many unique advantages of using the school setting to promote and protect pupil mental health [9]. First, most lifetime disorders begin during the schooling years [10], which suggests that schools may be an ideal setting for early identification and intervention. Second, schools have access to most children, meaning that they are an important component of any public health approach to address child mental health difficulties [11-14]. Third, schools benefit from prolonged engagement with pupils, which can facilitate the implementation of mental health promotion and prevention strategies as well as support and interventions for pupils with identified mental health needs [12]. Finally, mental health support in schools is often more accessible to families than other types of support [15].

However, while school staff are increasingly expected to support children’s mental health [1], many do not feel prepared to do so [16-19] due in part to receiving limited training and supervision in this area [20]. Therefore, improving school staff’s confidence and preparedness are important considerations for supporting them in taking an expanded role in pupil mental health [21]. Most schools offer some form of mental health training [22,23], but many staff members believe that they could benefit from additional training [18-20,24-26]. One area where staff training may be particularly beneficial is the identification of and first response to pupils who have mental health difficulties or who are believed to be at increased risk of developing them. However, although there is evidence suggesting that school staff, parents, and practitioners see such training as an acceptable, feasible, and potentially useful way to support pupil mental health [20,27-29], empirical evidence for the effectiveness of such training is limited and focuses primarily on intermediate outcomes (eg, staff knowledge and confidence) rather than downstream outcomes (eg, accurate identification, access to support, and mental health outcomes) [30,31]. Furthermore, there are several potential barriers to implementing training programs in schools, including time, cost, and resource requirements [28].

### *At-Risk for Elementary School Educators*: A Brief, Interactive Web-Based Training

Training programs that address these barriers may be beneficial for supporting schools in identifying and responding to pupil mental health difficulties. Brief, interactive web-based training programs are a particularly promising avenue as they have the potential to be more affordable, flexible, and scalable than other training formats. One such training is *At-Risk for Elementary School Educators* (hereinafter, *At-Risk*), a virtual simulation-based program developed by the American company Kognito [32]. The program, which has been completed by >125,000 teachers in the United States, aims to improve pupil mental health by “[building] awareness, knowledge, and skills about mental health, and [preparing] users to lead real-life conversations with pupils, parents, and caregivers about their concerns and available support” [33].

The program addresses many common implementation barriers to school-based mental health training. For example, *At-Risk* only requires approximately 1 hour to complete, which is much shorter than many other available training programs [31,34]. This comparatively low time commitment may address the concern that training programs are overly time intensive and, thus, make the training more feasible for busy schools [28,34,35]. The web-based format of *At-Risk* may also address concerns about school-based mental health programs being resource intensive [28]. Nearly all school mental health training programs documented in the literature are face-to-face sessions led by external facilitators [34,36], with only a few examples of web-based training [37-39]. For schools with limited budgets, programs requiring external facilitators can prove unsustainable and have limited scalability. In terms of financial resources, the costs of *At-Risk* vary depending on the number of licenses purchased, but the maximum cost is approximately £22 (US $30) per user, a price point that is feasible for many UK schools. In the United States, there have been many examples of bulk purchases at the district or state level that have made the training even more affordable per teacher. In many areas, the training is even free at point of use due to state- or district-wide licensing agreements [40].

To date, 3 randomized studies have examined the effectiveness and acceptability of *At-Risk* among samples of American teachers [17,41] and teachers in training [42] across school years. Each study found high satisfaction ratings, with between 75% and 85% of participants rating the training as useful, well constructed, relevant, and easy to use, and nearly all (88%-95%) reporting that they would recommend it to colleagues. The training also improved teachers’ self-rated preparedness, self-efficacy, and likelihood of identifying and discussing concerns about pupils’ mental health and referring them to appropriate support when needed. These improvements were reflected in the teachers’ behaviors—compared with teachers in the control group*,* those who completed *At-Risk* self-reported significantly more helping behaviors (eg, identifying psychological distress, discussing concerns with pupils and parents, and consulting with parents about options for care and support) and gatekeeping behaviors (ie, connecting pupils with care and support) after the training and at 3 months after the training. The findings of these studies indicate that *At-Risk* may help improve teachers’ ability to identify and respond to pupil mental health needs and lead to positive behavior change in terms of discussing concerns and facilitating access to care and support.

### *At-Risk* in a UK Context: Considerations for Transportability

These 3 studies suggest that *At-Risk* may be a promising intervention for improving children’s mental health; however, there is still much to be learned about the training’s effectiveness, feasibility, and acceptability. Furthermore, to date, no evaluation of the training has been conducted outside the United States. There is increasing focus on the influence of context on the effectiveness of complex interventions [43-48], and while some interventions have shown success in terms of transportability [48], other interventions that have evidence of effectiveness in one context have demonstrated null or even negative effects in another [46]. Furthermore, information that could inform “transportability” is often not collected as part of evaluations [44], making it difficult to determine the likelihood of success in a new setting.

There are many contextual differences between the United States and the United Kingdom that could mean that school-based interventions developed in one country may not translate well to the other. Cross-country differences in education systems and (mental) health services are particularly relevant to this study. Differences in the education system include the length and content of initial teacher training, the number and roles of teaching assistants (TAs), and school funding structures. There are also key differences in the structure and availability of school-based mental health provision. In the United States, schools often have staff whose sole or at least main responsibility is mental health, such as school psychologists. While these roles are becoming more common in the United Kingdom with the implementation of the Green Paper recommendations [1], in most UK primary schools, mental health is included within the broader roles of the special educational needs coordinator (SENCo) and pastoral team. Finally, differences in the wider health care systems across the countries also mean that the process and outcomes of external referrals to specialist mental health services vary across settings, another fact that may influence the transportability of school-based interventions such as *At-Risk.*

### This Study

Given these uncertainties regarding intervention transportability, additional evaluation of *At-Risk* is needed to understand whether it is a potentially useful and feasible tool to improve the identification of and response to mental health difficulties in UK primary schools. To explore the potential value of the training in this new context, we conducted a mixed methods feasibility study of *At-Risk* in 6 UK primary schools covering pupils aged 4 to 11 years*.* We aimed to examine the influence of *At-Risk* on staff confidence and preparedness, identification of pupils with mental health difficulties or increased risk of developing mental health difficulties, mental health support outcomes for identified children, and intervention acceptability and practicality.

## Methods

### Study Design

We used a mixed methods, nonrandomized, pretest-posttest study design to explore the feasibility of *At-Risk* in UK primary schools. While feasibility studies are acknowledged as a key stage of intervention design and evaluation [49,50], there is no universally agreed-upon definition of a feasibility study [50,51]. Therefore, we focused on 3 criteria from the guidance by Bowen et al [52]: acceptability, practicality, and limited effectiveness testing.

### Intervention: *At-Risk for Elementary School Educators*

**Figure 1:**
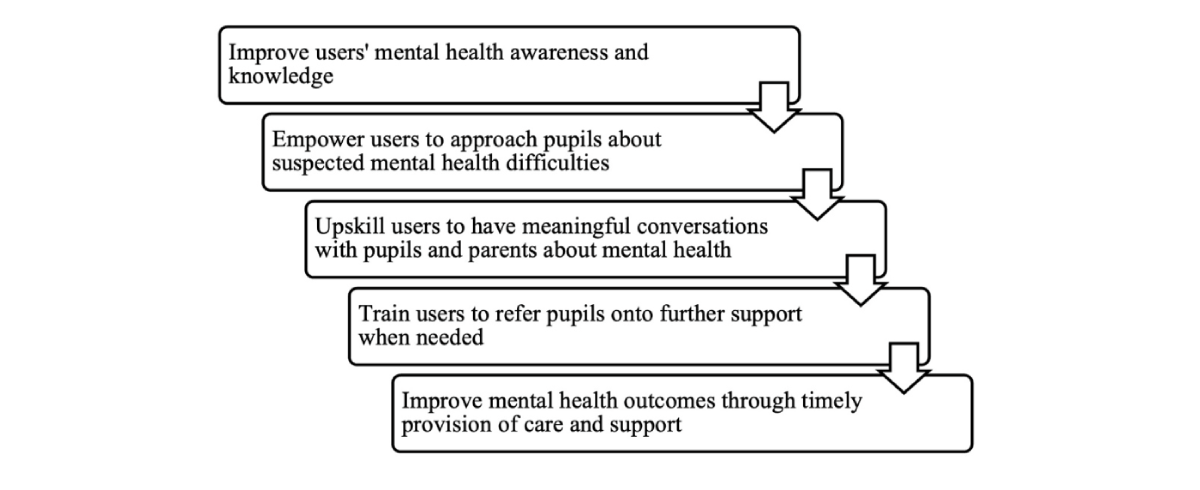
Theory of how the *At-Risk for Elementary School Educators* training may improve school staff’s recognition of and response to pupil mental health difficulties.

*At-Risk* is a web-based training that is delivered individually and requires only a log-in and internet connection. Using a simulation-based teaching model, the training aims to (1) improve mental health awareness and knowledge, (2) empower users to approach pupils about what they have noticed, (3) impart skills to have meaningful conversations with pupils and parents, and (4) train users to refer pupils to further support. The diagram in Figure 1 illustrates how the training might lead to improved mental health outcomes for pupils.

The simulation begins with an introduction by a virtual coach, who defines and explains how to recognize the warning signs of psychological distress and specific mental health difficulties and provides guidance and practical advice for discussing and acting upon concerns. Users then practice 2 virtual scenarios. The first scenario involves a fifth-grade (UK Year 6; ages of 10-11 years) teacher speaking with the parent of a pupil showing signs of behavioral difficulties. The second involves a third-grade (UK Year 4; ages of 8-9 years) teacher speaking with a pupil showing signs of emotional difficulties. During the conversations, users choose what to say via drop-down menus organized into categories (eg, “bring up concerns” or “ask a question”) and phrases (eg, “Mia sometimes seems a little agitated in class”). Throughout the conversation, users receive feedback through a “comfort bar” (based on how the pupil or parent perceives the conversation), opportunities to “see” the thoughts of the pupil or parent, and suggestions from the virtual coach.

Importantly, there is no one “right” way to conduct the conversations, and several approaches can lead to a positive outcome. Throughout the conversation, users can “undo” actions to backtrack after receiving an undesirable response or to explore what the response would have been had they chosen another option. At the end of each conversation, the pupil or parent provides feedback on the conversation. The training finishes with a short segment on connecting pupils with further support.

For this feasibility study, we used an unmodified version of the training (ie, the standard training designed for American schools, not tailored to the UK context) provided free of cost by Kognito. The potential need for adaptation and tailoring was an important consideration that we explicitly examined as part of our exploration of the acceptability and practicality of *At-Risk* in this new setting.

### Recruitment

#### Schools

We originally sought to purposively sample 5 primary schools from Cambridgeshire or Norfolk that (1) had a higher-than-average proportion of pupils eligible for free school meals or (2) were located in an area in the top tertile of deprivation as measured using the Index of Multiple Deprivation [53], which we calculated with the publicly available Schools, pupils and their characteristics data [54]. We emailed headteachers, SENCos, and mental health leads from 131 candidate schools in September and October 2019 about participating in the study. To increase recruitment, we contacted additional schools in January 2020 for a study start date of March 2020. However, the study was suspended in March 2020 due to the in-person school closures associated with the onset of the COVID-19 pandemic. As some of the participating schools dropped out due to the pandemic, we reopened recruitment for a January 2021 study start date. In this round, we did not restrict participation by the 2 deprivation criteria described previously (ie, free school meal eligibility and Index of Multiple Deprivation), so any UK-based mainstream primary school was eligible to participate. The January 2021 start date was again delayed by the pandemic, but there was no subsequent recruitment.

#### Teachers and TAs

Schools were responsible for recruiting individual teachers and TAs to participate in the training. We encouraged schools to invite all teachers and TAs to participate, but schools made a variety of decisions in this regard. Three schools (schools D, E, and F) had all staff complete the training during inset (in-service training) days or other designated times, 2 schools (schools A and C) had staff volunteer to participate, and 1 school (school B) selected 2 to 3 staff members in each year group to participate.

### Measures and Materials

#### School Characteristics

The characteristics of the participating schools, including school type, school sex (ie, whether they were single or mixed sex), urbanicity, head count, area-level deprivation, level of free school meal eligibility, ethnic composition, and proportion of pupils with special educational needs, were obtained from publicly available data from the Department for Education [54,55].

#### Teacher and TA Identification Form

The purpose of the Teacher and TA Identification Form (Multimedia Appendix 1) was to understand which pupils participants would identify as having mental health difficulties or an increased risk of developing mental health difficulties. As systematic reviews in this area have identified no suitable questionnaires [28,30], we developed a bespoke questionnaire, which was reviewed by a school staff advisory group to ensure accuracy and relevance. The questionnaire begins with instructions, including explanations and examples of what is meant by “mental health difficulties or risk for mental health difficulties.” Full definitions are provided in Multimedia Appendix 1, but in brief, “mental health difficulties” are described as “behavioural and social-emotional problems” regardless of formal diagnosis, and “risk for mental health difficulties” is described as experiences that increase the chance of a child developing mental health difficulties in the future.

For all pupils in their class, participants first indicated whether they thought a pupil had mental health difficulties or increased risk. If yes, they answered 9 subsequent questions about mental health support outcomes. The first four outcomes were about communication of concerns, namely whether they had (1) formally documented their concerns with the school, (2) communicated concerns to the SENCo, pastoral care lead, or mental health lead, (3) communicated concerns to another member of the school staff, or (4) communicated concerns to the child or their parents. The next five outcomes pertained to the provision of mental health support, namely whether the pupil (5) received in-class support; (6) received in-school support or had an in-house support plan; (7) had documented social, emotional, and mental health (SEMH) status (a type of special educational need focused on mental health difficulties); (8) had been referred to external mental health services; or (9) had access to external mental health services.

#### Strengths and Difficulties Questionnaire

The teacher-report Strengths and Difficulties Questionnaire (SDQ) [56-59] served as the comparator for findings about teachers’ and TAs’ identification of pupils. The SDQ includes 25 positive and negative psychological attributes across 5 scales: emotional symptoms, conduct problems, hyperactivity/inattention, peer relationship problems, and prosocial behavior. The first 4 scales add up to a Total Difficulties Score (0-40, with higher scores representing greater difficulties). The SDQ has demonstrated acceptable psychometric properties in primary school samples [60]. It is important to note that the SDQ is not an exact comparator as it measures a narrower concept than the Teacher and TA Identification Form (which also includes *increased risk*). However, this comparison could potentially yield valuable information regarding feasibility*.*

#### Pre- and Posttraining Surveys

Kognito uses pre- and posttraining surveys to assess their training. These surveys (based on the validated Gatekeeper Behavior Scale [61]) explore teachers’ self-efficacy in identifying and responding to mental health difficulties and whether their attitudes, self-efficacy, or practice have changed since completing the *At-Risk* training. The posttraining survey also includes questions on perceptions of the training’s impact. We independently (ie, with no input from Kognito) reviewed the merits of these questionnaires and decided to use them in this study because (1) they covered relevant and useful concepts related to our aims and (2) using them increased comparability to the other 3 US-based studies of *At-Risk*. We slightly adapted the surveys to make them more relevant to the UK context (Multimedia Appendix 2).

#### Interview Schedules

For the pretraining interviews with SENCos and mental health leads, we developed a topic guide about current practice (Multimedia Appendix 3) with the specific purpose of creating Mental Health Resource Maps for each school (refer to the *Procedures* section). The main topics pertained to formal and informal procedures for when staff members suspect that a child might have mental health difficulties or increased risk, as well as the types of support available.

For the posttraining interviews with teachers, TAs, and strategic stakeholders (ie, those with key leadership roles, including senior leadership teams [SLTs], school governors, and SENCos and mental health leads), we developed 3 separate topic guides (Multimedia Appendix 3), which were informed by our research questions, systematic reviews [28,30], and the Consolidated Framework for Implementation Research [62,63]. For teachers and TAs who completed *At-Risk* and strategic stakeholders, interview topics included the acceptability of the training, the practicality of implementing it in schools, the utility of further refinement and testing, possible harms associated with the training (if any), and suggestions for adaptations. For teachers and TAs who did not complete the training, topics included reasons for not completing it, barriers to acceptability and practicality, and suggestions for adaptations.

### Procedures

#### Interviews With SENCos and Mental Health Leads

We conducted a pretraining interview with each school’s SENCo or mental health lead to develop a “Mental Health Resource Map” with information on referral processes and available support. These maps served an ethical purpose by ensuring that pupils identified as potentially having mental health difficulties would have the best possible chance of being linked to care and support.

#### Completing *At-Risk*

Schools’ timelines for the study varied due to the pandemic and other commitments. School D completed the training in December 2020; school E completed the training in March 2021; schools B, C, and F completed the training in May 2021; and school A completed the training in June 2021. At baseline (T1), participants completed a Teacher and TA Identification Form and the pretraining survey. They then completed the *At-Risk* training. We encouraged schools to designate specific time for the training, which 3 schools (schools D, E, and F) did. One week after training (T2), participants were asked to complete a second Teacher and TA Identification Form and the posttraining survey. Three months after the training or at the end of the school year (whichever came first; T3), participants completed a third Teacher and TA Identification Form as well as SDQs for all pupils. All questionnaires were completed on the University of Cambridge Qualtrics platform (Qualtrics International Inc).

#### Feedback Provision

After T2, we provided all SENCos and mental health leads but not teachers or TAs with feedback regarding which children had been identified as having mental health difficulties or increased risk. After T3, we provided SDQ scores for each child as well as whole-class distributions (where available). This feedback was provided to ensure the ethical conduct of the study.

#### Interviews With Teachers, TAs, and Strategic Stakeholders

We aimed to recruit at least 3 teachers or TAs who completed the training per school, 3 to 5 teachers or TAs who had not completed the training across all schools, and up to 3 strategic stakeholders per school for posttraining semistructured interviews. Schools contacted staff members directly with an invitation to complete a virtual interview.

### Analysis

#### Quantitative Outcomes

##### Analytical Samples

For the main analysis, participants were included if they (1) completed at least the pretraining (T1) questionnaires and the training itself and (2) had what we judged to be a typical number of children they regularly worked with. For the latter criterion, given that the average UK primary school class size is approximately 27 to 28 pupils [64], we excluded teachers and TAs who worked with <10 children (as we suspected this would not be a random selection of pupils and would therefore influence aggregate identification rates) and those who worked with >60 children (as we believed that it would be difficult for a teacher or TA to know >2 classes’ worth of children well enough to make accurate judgments about their mental health).

##### Teacher and TA Self-Efficacy and Preparedness

To assess teachers’ and TAs’ preparedness, self-efficacy, and perceptions of training impact, we calculated the absolute and relative frequencies of responses to the pre- and posttraining surveys. Participants were eligible for inclusion in this analysis only if they had pretraining (T1) data.

##### Identification Outcomes

On the basis of the Teacher and TA Identification Forms, we calculated the number and percentage of pupils in each class whom teachers and TAs perceived as having mental health difficulties or increased risk at each time point. We summarized these across all participants using medians and IQRs.

We then calculated SDQ scores, which we compared with responses from the Teacher and TA Identification Form by calculating (1) the median and IQR for the percentage of children identified by participants who *did not* have elevated SDQ scores and (2) the median and IQR for the percentage of children with elevated SDQ scores who *were not identified by* participants. To be included in these analyses, participants had to have completed all 3 time points. For the first outcome, they had to have completed an SDQ for *all children they identified in the Teacher and TA Identification Form*. For the second outcome, they had to have completed SDQs for at least 80% of their class. Where it was possible to match pupil IDs between teachers and TAs, we pooled SDQ data such that, if one participant did not meet the inclusion criteria themselves, they could still be included if the SDQ data were available from another staff member working with the same children.

##### Mental Health Support Outcomes

Finally, for each time point, we calculated medians and IQRs for the proportion of identified children with each of the 9 mental health support outcomes (refer to the *Teacher and TA Identification Form* section for the outcomes)*.*

##### Sensitivity Analyses

We also conducted 2 post hoc sensitivity analyses. The first sensitivity analysis excluded all participants from school D. When we prepared feedback for school D (the first school to complete the training), we learned that most participants at the school had misinterpreted the Teacher and TA Identification Form. We edited the form and instructions accordingly to address this issue, but therefore, school D participants completed a slightly different form than the other schools. The second sensitivity analysis was a complete case analysis intended to explore observed differences in outcomes according to whether participants had completed all 3 time points. For the analysis of outcomes pertaining to preparedness, self-efficacy, and perceptions of training impact, we included all participants who completed the surveys *at least* at T1 and T2.

##### Statistical Analysis

For all quantitative outcomes, we focused on preliminary, descriptive comparisons across the 3 time points and did not perform any formal hypothesis testing. This aligns with established recommendations for feasibility studies, which generally lack the statistical power necessary for a clear interpretation of hypothesis-testing results [65-68]. We conducted all quantitative analyses in R (version 4.0.3; R Foundation for Statistical Computing) [69] except for the comparison of Teacher and TA Identification Forms and SDQ scores, for which we used Microsoft Excel (Microsoft Corp). We created all plots using the *ggplot2* [70] and *likert* packages [71]. To score the SDQs, we used the freely available R code on the Youthinmind website [72].

#### Qualitative Outcomes

We considered 3 analysis approaches for the interview and qualitative questionnaire data: content analysis [73], framework analysis [74], and reflexive thematic analysis [75,76]. We initially decided to use content analysis for the survey comments and reflexive thematic analysis for the interviews; however, as we familiarized ourselves with the data, we realized that there was significant overlap between the survey comments and interviews and decided that analyzing them separately was not a useful distinction. As our main aim was to generate insights into the program and its future potential, we decided to use the 6-phase reflexive thematic analysis by Braun and Clarke [76] for all qualitative data due to its flexibility and ability to generate themes both inductively and deductively. ES developed the initial themes, and MF and EH helped clarify and enrich them. ES and MF worked together to name and refine the themes before the final write-up. We managed and coded all qualitative data in ATLAS.ti (version 9.1.3; ATLAS.ti Scientific Software Development GmbH) and additionally created manual thematic maps to better visualize and understand patterns between our data.

### Ethical Considerations

This study was approved by the University of Cambridge Psychology Research Ethics Committee (PRE 2019.076). We obtained active informed consent from all teachers and TAs who took part in the study. We used an opt-out model for parental consent whereby parents received (directly from the schools via their preferred communication routes) an information sheet detailing study aims, procedures, how data would be used, and the right to opt their child out of participation. Parents had 2 weeks to opt their child out of the study by returning a hard copy of the opt-out form or emailing or calling the school. Schools kept track of all opt-outs and instructed teachers and TAs not to include these children in their forms. All quantitative data were collected using anonymous pupil and staff identifiers generated by the participating schools, and all qualitative data were deidentified before analysis, with identifiable information stored on secure servers at the University of Cambridge. Teachers and TAs received £20 (approximately US $28) vouchers for completing the training and questionnaires for at least 2 of the 3 time points and an additional £10 (approximately US $14) for taking part in an interview. School staff members who created the anonymous identifiers received £10 (approximately US $14) vouchers to thank them for their time.

## Results

### Participants

#### Schools

A total of 6 schools participated in this study (Table S1 in Multimedia Appendix 4 [40]). Among these 6 schools, there were 4 (67%) from Cambridgeshire and 1 (17%) each from Greater London and Merseyside; 5 (83%) were located in urban areas and 1 (17%) was located in a rural area. All but 1 school (5/6, 83%) were situated in areas of above-average deprivation, and 50% (3/6) of the schools had a higher-than-average proportion of pupils eligible for free school meals. In total, 67% (4/6) of the schools had a high proportion of White pupils (>80%), and 33% (2/6) of the schools were more diverse, with approximately 20% of pupils from Black, Black British, Caribbean, or African backgrounds (school B) or Asian or Asian British backgrounds (school E).

#### Teachers and TAs

A total of 108 teachers and TAs completed the T1 questionnaires and the training itself, 89 (82.4%) completed the T2 questionnaires, and 70 (64.8%) completed the T3 questionnaires (Table 1), with 54 (50%) having completed all 3. After excluding those teachers and TAs who did not meet the inclusion criteria for the analyses, the final analytical samples were as follows:

1. Main analysis of identification and mental health support outcomes: n=97 at T1, n=75 at T2, and n=57 at T3.
2. Main analysis of preparedness, self-efficacy, and training impact outcomes: n=107 at T1 and n=83 at T2.
3. Main analysis comparing identification outcomes with SDQ scores: n=28 and n=25 (refer to the following section).
4. Complete case sensitivity analysis: n=51 at T1, T2, and T3.
5. Sensitivity analysis excluding all teachers and TAs from school D: n=70 at T1, n=54 at T2, and n=41 at T3.

Compared with the 2019-2020 national workforce statistics for teachers and TAs working in state-funded nursery and primary schools [77], our sample had a similar proportion of women (81/89, 91% in our sample vs 90.9% nationally) and a slightly higher proportion of White staff members (82/89, 92% in our sample vs 90.5% nationally).

A total of 7.4% (8/108) of school staff members from 67% (4/6) of the schools completed an interview (Table 2).

**Table 1:** Characteristics of participating teachers and teaching assistants (TAs) (N=89^a^).

Characteristics		Values
**Gender, n (%)**		
	Woman	81 (91)
	Man	6 (7)
	Missing	2 (2)
Age (y), mean (SD)^b^		40.3 (10.9)
**School, n (%)**		
	A	7 (8)
	B	15 (17)
	C	2 (2)
	D	28 (31)
	E	22 (25)
	F	15 (17)
**Position^c^, n (%)**		
	Teacher	42 (47)
	TA (including higher-level TAs)	40 (45)
	SENCo^d^, mental health champion, or mental health lead	3 (3)
	School counselor or psychologist	1 (1)
	Member of senior leadership team	6 (7)
	Prefer not to say	1 (1)
	Missing	1 (1)
Years of experience, mean (SD)^e^		12.2 (7.7)
**Ethnicity, n (%)**		
	Asian or Asian British	2 (2)
	Mixed or multiple ethnic groups	1 (1)
	White	82 (92)
	Other ethnic group	2 (2)
	Prefer not to say	1 (1)
	Missing	1 (1)

^a^N=89 because this information was collected only at T2.

^b^N_NA_=4 (number with missing data for this question).

^c^Percentages add up to >100 because some participants had multiple roles.

^d^SENCo: special educational needs coordinator.

^e^N_NA_=7 (number with missing data for this question).

**Table 2:** Characteristics of the interviewees (n=8).

Interviewee ID	School	Main role or roles
1	B	Assistant headteacher for mental health and well-being, PSHE^a^ lead, and class teacher (Reception)
2	B	Assistant headteacher and class teacher (Year 4)
3	E	Class teacher (Year 6)
4	E	SENCo^b^
5	F	SENCo (note: oversaw training but did not complete it)
6	C	Mental health lead and TA^c^ (across years)
7	E	Mental health champion and HLTA^d^ (Year 5 and 6)
8	F	TA (Early Years Foundation Stage)

^a^PSHE: Personal, Social, Health and Economic.

^b^SENCo: special educational needs coordinator.

^c^TA: teaching assistant.

^d^HLTA: higher-level teaching assistant.

### Findings

#### Teacher and TA Self-Efficacy and Preparedness

**Figure 2:**
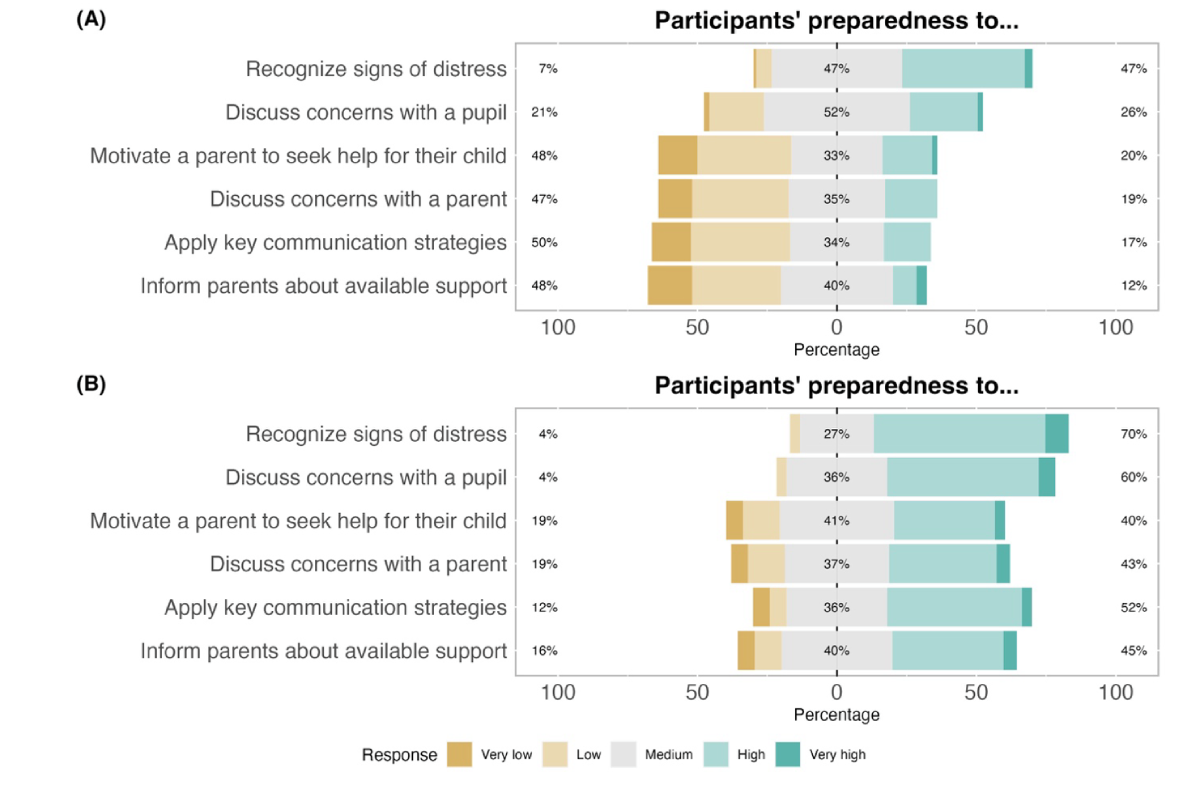
School staff preparedness to identify and respond to pupil mental health difficulties before and 1 week after completing the *At-Risk for Elementary School Educators* training (n=107 at T1; n=83 at T2). (A) Before training; (B) After training.

**Figure 3:**
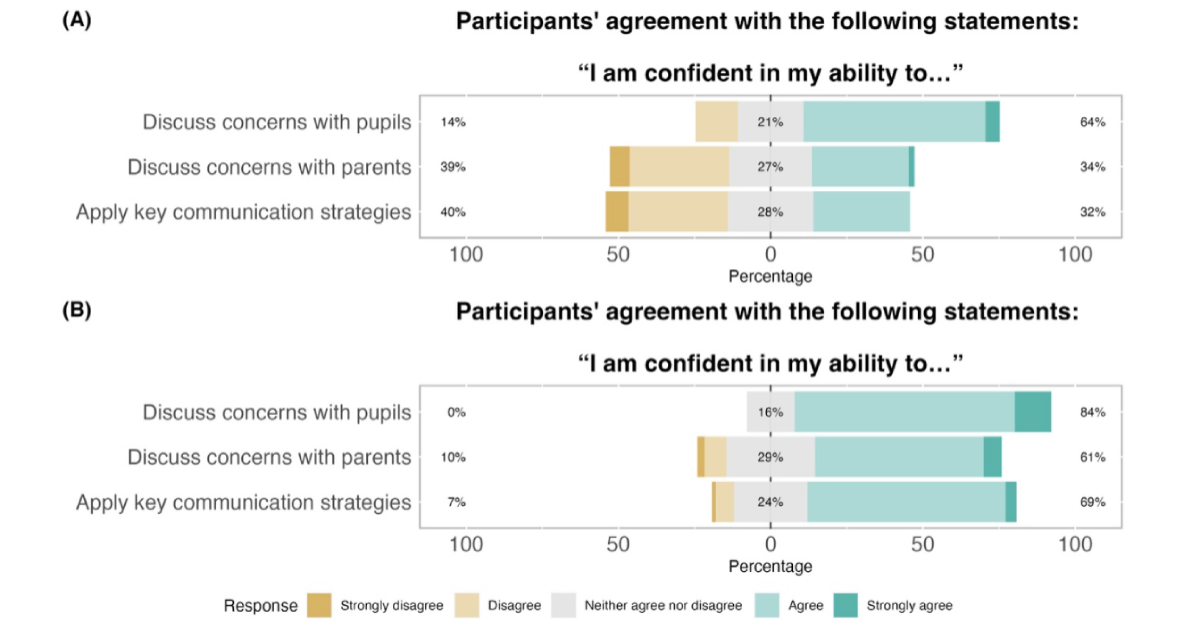
School staff self-efficacy to identify and respond to pupil mental health difficulties before and 1 week after completing the *At-Risk for Elementary School Educators* training (n=107 at T1; n=83 at T2). (A) Before training; (B) After training.

**Figure 4:**
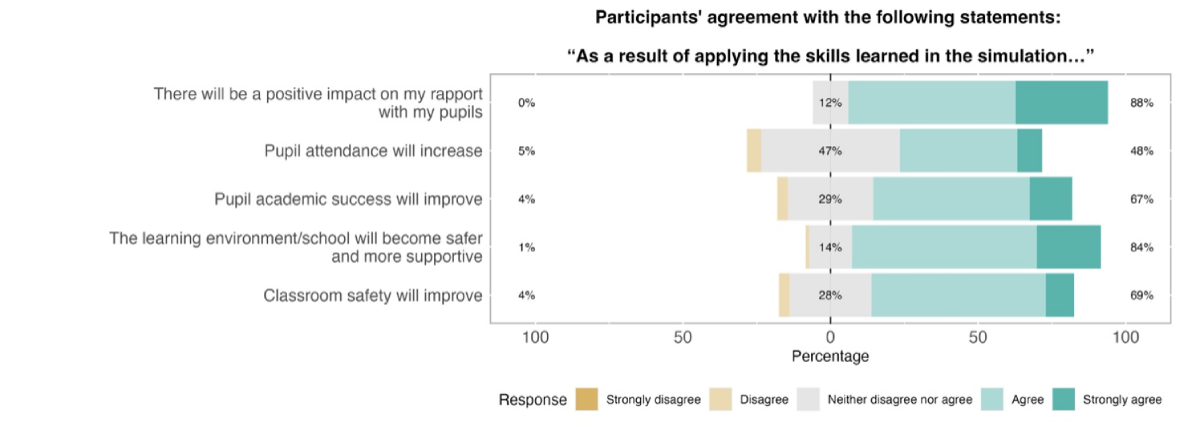
School staff perceptions of the impact of the *At-Risk for Elementary School Educators* training, assessed 1 week after completing it (n=83).

Pretest-posttest changes suggested that participating in the training was beneficial for the staff and that they had positive perceptions of the training. Findings regarding preparedness (Figure 2) suggest improvements across all domains of recognizing and acting upon concerns about pupils’ mental health, particularly in terms of using key communication strategies and working with parents. Findings regarding self-efficacy (Figure 3) suggest that participants were more confident in their abilities to discuss their concerns about pupils’ mental health after the training than before. Again, the largest changes were observed in discussing concerns with parents and applying key communication strategies. Finally, findings regarding teachers’ and TAs’ perceptions of the impact of applying the skills of the training (Figure 4) suggest that they were generally positive about the possible effects of the training on pupil outcomes (ie, attendance and academic success), teacher-pupil rapport, and the classroom environment. The results from the complete case analysis (Multimedia Appendix 5) were nearly identical to those of the main analysis (all differences were ≤3 percentage points in magnitude).

#### Identification Outcomes

In terms of how many pupils were identified as having mental health difficulties or increased risk, participants identified similar proportions of their pupils before and immediately after the training and then fewer over time. The median percentage of pupils whom participants believed had mental health difficulties or increased risk was 10% (IQR 6.7%-18.2%) at T1, 10% (IQR 4.5%-16.7%) at T2, and 7.4% (IQR 5.0%-16.7%) at T3. The directions of change were similar for both sensitivity analyses (whereby teachers and TAs identified *fewer* children over time), with slight differences. For the sensitivity analysis excluding school D (Multimedia Appendix 6), the percentages were slightly (approximately 2 percentage points) higher. For the complete case analysis, the decrease was also notable 1 week after the training, decreasing from 10% (IQR 6.7%-17.3%) at T1 to 8% (IQR 3.9%-16.7%) at T2 and 7.4% (IQR 5.7%-16.7%) at T3.

In terms of the accuracy of identification, it seems that teachers and TAs became slightly more accurate over time in comparison to pupils’ SDQ scores (although it is important to acknowledge the limitations described in the *Methods* section regarding questionnaire comparability). The median percentage of children identified by participants who *did not have elevated SDQ scores* was 40% (IQR 0%-50%) at T1, 27.2% (IQR 0%-50%) at T2, and 25% (IQR 0%-50%) at T3. The median percentage of children with elevated SDQ scores *who were not identified by participants* was 68.8% (IQR 42.9%-87.5%) at T1, 66.7% (IQR 50%-88.2%) at T2, and 57.1% (IQR 33.3%-87.5%) at T3. In the sensitivity analysis excluding school D, the results were similar (typically within 5 percentage points); one small difference was that the median percentage of children identified by teachers and TAs who did not have elevated SDQ scores was 0% (IQR 0%-50%) at T2. The results of the complete case analysis were identical to those of the main analysis.

#### Mental Health Support Outcomes

**Figure 5:**
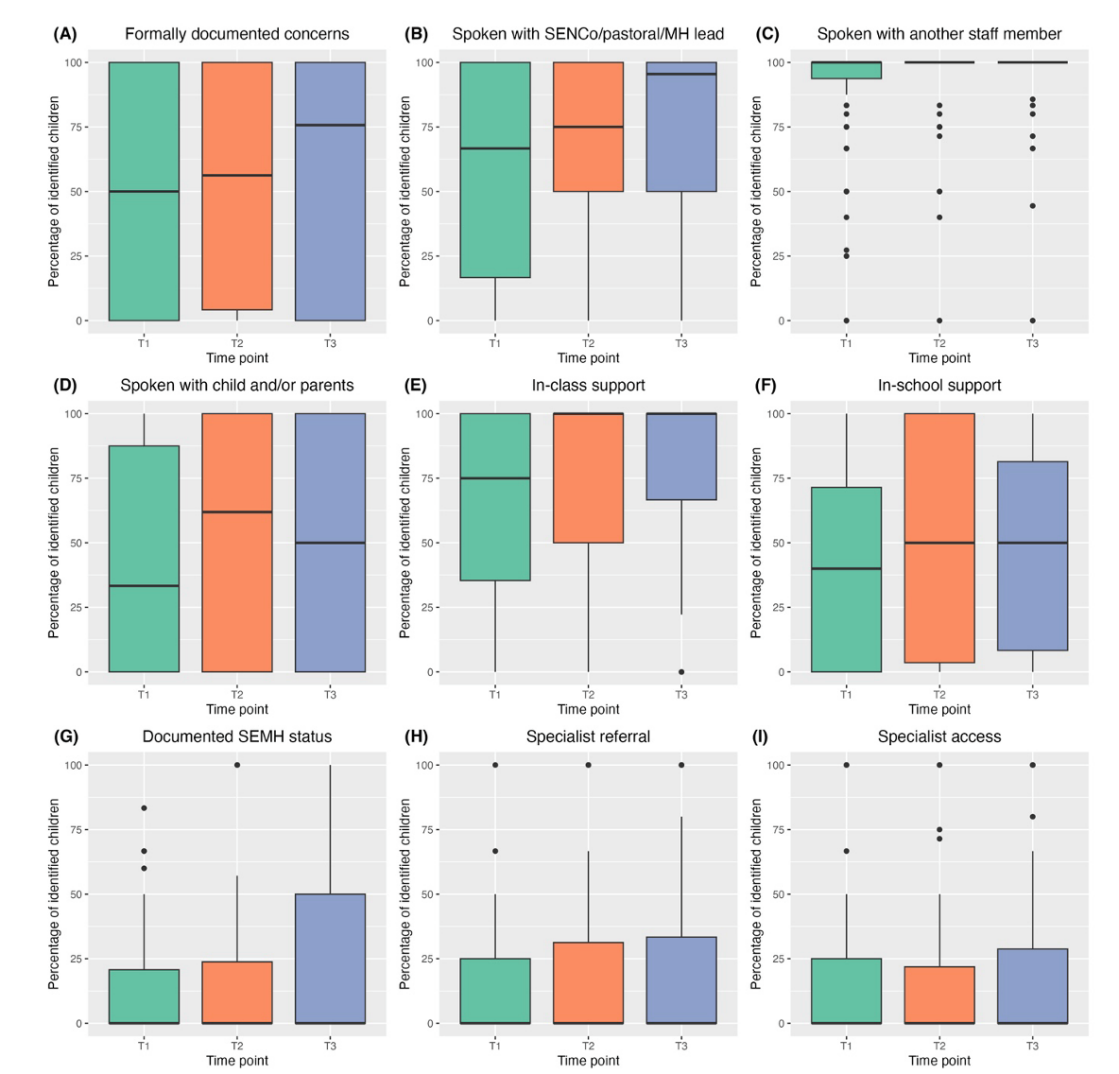
Mental health support outcomes assessed before (T1), 1 week after (T2), and approximately 3 months after (T3) completing the *At-Risk for Elementary School Educators* training. Staff-related outcomes include (A) formally documenting concerns with the school; (B) communicating concerns to the special educational needs coordinator (SENCo), pastoral care lead, or mental health (MH) lead; (C) communicating concerns to another member of the school staff; and (D) communicating concerns to the child or their parents. Pupil outcomes include (E) receiving in-class support; (F) receiving in-school support or having an in-house support plan; (G) having documented social, emotional, or MH (SEMH) status; (H) having a referral to external MH services; and (I) having access to external MH services. These outcomes exclude participants who were not concerned about any child (n=2 at T1; n=5 at T2; n=5 at T3).

Overall, the findings suggest that the training may be beneficial for facilitating conversations and access to school-based support (but not external support) for pupils with identified mental health difficulties or increased risk. Figure 5 presents the findings for the 9 mental health support outcomes among identified children across the 3 study time points. As with before the training, there was typically a wide variation in outcomes.

A comparison across time points suggests that participants formally documented their concerns and spoke with the SENCo, pastoral lead, or mental health lead for a greater proportion of identified pupils after the training than before. For example, at T1, teachers and TAs documented concerns for a median of 50% (IQR 0%-100%) of identified pupils; this increased to 56.3% (IQR 4.2%-100%) at T2 and 75.7% (IQR 0%-100%) at T3. The equivalent statistics for speaking with the SENCo, pastoral lead, or mental health lead were a median of 66.7% (IQR 16.7%-100%) at T1, 75% (IQR 50.0%-100%) at T2, and 95.5% (IQR 50.0%-100%) at T3. There was no change in speaking with another staff member, but this was because nearly all participants did so across all time points. Finally, the percentage of pupils whom teachers and TAs spoke with (or whose parents they spoke with) also increased after the training, with a median of 33.3% (IQR 0%-87.5%) at T1, 61.9% (IQR 0%-100%) at T2, and 50% (IQR 0%-100%) at T3.

A comparison across time points also suggests increases in school-based support for identified children after the training compared with before. The median percentage of pupils identified by teachers and TAs who received in-class support increased from 75% (IQR 35.4%-100%) at T1 to 100% at T2 and T3 (IQR 50%-100% and 66.7%-100%, respectively). There was a more modest increase in the receipt of in-school support or in-house support plans, with a median of 40% (IQR 0%-71.4%) of identified pupils receiving them at T1 compared with 50% at T2 and T3 (IQR 3.6%-100% and 8.3%-81.4%, respectively). There was very little change in documented SEMH status or referral or access to specialist mental health services. For each of these outcomes, the median percentage of identified pupils was 0% across time points.

The findings from the sensitivity analyses were similar to those of the main analysis in terms of direction, although improvements across time in the complete case analysis (Multimedia Appendix 5) tended to be more modest than for the main analysis.

#### Acceptability and Practicality

##### Quantitative Findings

Quantitative data from the posttraining survey showed that participants were generally positive about the training. Of the 83 participants who completed the survey, 53 (64%) rated it as “good” and 13 (16%) rated it as “very good.” An additional 17% (14/83) rated it as “fair,” 2% (2/83) rated it as “poor”, and 1% (1/83) as “very poor.” A total of 84% (70/83) of the teachers and TAs said that the scenarios in the training were relevant to them. Finally, most participants (74/83, 89%) would recommend the training to other educators.

##### Qualitative Findings

Qualitative data also suggested that school staff generally found the training practical and acceptable. We generated three themes from our survey and interview data:

1. Individual fit: positive perceptions, self-efficacy, and change.
2. Institutional fit: alignment with school values and context.
3. Taking it forward: improvements and implementation.

Additional findings on possible harms are presented in Multimedia Appendix 7.

##### Individual Fit: Positive Perceptions, Self-Efficacy, and Change

In general, participants perceived the program to be a “good fit” with their personal philosophies and practice. Regarding the training itself, many appreciated the included scenarios, particularly in terms of their relevance to their practice. The format of the training—primarily that it was web-based and required active role-play—was also viewed as useful, engaging, and novel and might have contributed to its perceived usefulness. For example, one teacher commented:

The interactive elements of the training were brilliant and something which I have never encountered before!

Survey respondent (SR) 56; school E

One teacher and well-being lead described:

I think it definitely made you think. [...] you had to really think about what was being said and the response that you would give, reflecting back on sort of the knowledge that they’d given you beforehand, so I thought that was good.

Interviewee 1

Other participants suggested that opportunities to practice skills during the training improved the likelihood of using those skills in day-to-day practice.

Participants also believed that they had learned a lot from the training, especially in terms of *skills and strategies.* These included but were not limited to the skills within the *At-Risk* “EASING” strategy (check your Emotions, Ask for permission, be Specific, use *I* statements, keep it Neutral, and show Genuine curiosity). Importantly, there was evidence that participants had also *applied* new skills. Several participants described having new conversations with pupils or parents facilitated by the skills and strategies from the training. For example, one teacher described:

It was that permission thing [...] I wanted to ask [a child] about his home life [...] and kind of he just cried and didn’t want to speak about it anymore, and then when I asked him if we were OK to talk about it, he said, “Actually no, because I think I’m going to cry again,” so then we left it. And then he came to me the following week, and [...] said, “Can we talk about it now?” [...] so actually me asking that, it was the wrong time for him to talk about it, he wasn’t ready, he would have just been emotional, and wouldn’t have been able to get his words out, and actually the week after, him coming to me and saying, “Can we have a little chat,” works perfectly [...] And now we’re more aware of his situation.

Interviewee 2

This skill seems to have enabled this pupil to have this conversation with the teacher in a manner (in terms of time, place, and identified person) that suited him. Other participants provided similar examples, referencing how skills from the training had facilitated better outcomes.

However, it is important to note that the perceived usefulness of the training varied. Most notably, some participants indicated that their previous training or role made the training less impactful. Illustratively, when asked how the training had impacted their practice, one TA responded:

Having previously received similar training, due to my role, I do not have any recent cases where the training would have changed the way I carried out discussions.

SR 60; school E

##### Institutional Fit: Alignment With School Values and Context

Sustainable school-based programs should also align with the values of the school more broadly. Participants often referenced the importance of schools’ prioritization of pupil mental health. For example, one teacher described:

[Mental health is] a conversation which is constantly ongoing and trying to constantly better our practices and make sure we’re looking after them as best as we can and spotting things as best we can as well.

Interviewee 3

This description demonstrates how prioritizing mental health can promote the critical evaluation of related school practices as well as the additional provision of training opportunities. In many cases, support from the SLT led to *formal* recognition of pupil mental health within school policies or plans. One strategic stakeholder explained:

I think because our school have well-being and mental health as such a focus, SLT are very supportive of doing things like this and they’re very accommodating. So when I said we had the training and people were going to have to take part in the training, it was very flexible, although they had other ones lined up, they were quite happy to move things around to make things work. And I think, the fact it is such a priority in our school definitely makes that easier.

Interviewee 1

In this school, mental health and well-being were one of three main school priorities. As indicated previously, direction setting from the SLT is key to ensuring momentum and impetus. However, as others noted, it is important that support from the SLT is genuine rather than being “just another tick box” (Interviewee 4) exercise.

Another facet of institutional fit pertained to the practical aspects of the training. Schools are time- and resource-limited settings, so mental health training needs to fit within this context. The format of *At-Risk,* especially its flexibility and relatively low time requirements, was viewed as beneficial, with comments such as “For the amount of time [...] I got a huge amount from it” (Interviewee 4). Others made direct comparisons with other training courses. For example, one higher-level TA had previously completed a 1-day, in-person training course with a similar purpose to that of *At-Risk.* While she preferred the in-person training, she listed the benefits of both types:

[In the in-person training] you can then query and question to your trainer, so you’ve got that interaction, so that obviously isn’t there, is it, on the computer one. [...] if I was looking from a management point of view, I would say, budgetary, I’m sure it’s cheaper [...] to use [At-Risk], not just cheaper as in [...] money, [...] but also cheaper in time [...] So probably if I was looking [...] with my management hat on, I would say the computer-based [training] would get the same message, or similar message, across for a wider audience for probably a cheaper cost.

Interviewee 7

In terms of efficiency, this participant highlighted the favorable input-to-output ratio of *At-Risk*, which could allow more staff members to participate in training. This quote also highlights that schools could use *At-Risk* flexibly. For example, schools might assign staff members to different training programs based on their roles and previous experience, with more intensive, in-person training for staff members with more significant mental health roles and *At-Risk* for those with fewer responsibilities or less experience.

##### Taking It Forward: Improvements and Implementation

Participants offered key insights into how to take the training forward in terms of both changes to the training itself and how best to implement it, primarily by tailoring it to the UK context. In terms of language, there was some reference to the American accent, but more so, participants highlighted the need to adapt some of the terminology and signposting resources to reflect UK support systems. They also made suggestions about additional training that could be useful with different topics (such as bullying) and age groups (particularly for younger children).

In addition to improvements and adaptations to the training itself, participants illustrated the importance of implementation. A common theme was that, to maximize impact, the training should include follow-up discussions or live workshopping. One teacher suggested:

I think some kind of “live” element to conclude the training—to have a “real” person to ask questions to as part of a group video chat could have been useful. Also, maybe to ask advice about particular scenarios that we may have found ourselves in in the past.

SR 56; school E

By facilitating greater engagement and critical thinking, a live element could enhance the impact of the training and potentially make *At-Risk* more acceptable to those who generally prefer face-to-face training. Participants indicated that someone internal, for example, the SENCo, would be best placed to lead a live element and would enable staff to practice role-playing based on situations and scenarios specific to each school.

There was also wide acknowledgment that any training had to lie within a strong support system. This began with having a clear referral pathway for identified concerns, which was viewed as important for facilitating access to support. In some cases, teachers and TAs were able to find new ways to support children after completing the training. However, in many cases, participants—and strategic stakeholders in particular—explained that support had not always been readily available. For example, one strategic stakeholder recounted what happened after the training:

A lot of them are people saying to me, “What are you going to do about it?” about different children. And I, because some of our support staff don’t know the sort of route for getting extra support, or they’re really shocked to find actually there’s nothing out there for these children...it’s about what we can do in school, and I think people have been really quite shocked about that. You know, they just presume I can make a phone call and these children will get face-to-face counselling.

Interviewee 5

This shows the importance of embedding the training within a wider support system, including collaboration with external agencies. However, many interviewees referenced the systemic issues that schools face in helping pupils access specialist support, particularly in terms of the high thresholds and long waiting lists that exist for many external services. While schools may be able to provide beneficial support for children, particularly for those with lower-level difficulties, this indicates an ongoing area of need for schools and their pupils.

## Discussion

### Summary of Findings

This study offers the first UK evidence for Kognito’s *At-Risk for Elementary School Educators*, extending findings from 3 US-based trials and providing needed evidence regarding the potential utility, acceptability, and practicality of brief, interactive web-based mental health training for school staff. Overall, the findings showed that *At-Risk* is a feasible means of improving the identification of and response to pupil mental health difficulties in UK primary schools. Quantitative findings showed that staff preparedness and self-efficacy in identifying and responding to mental health difficulties increased after the training. Identification rates did not increase (and, in fact, decreased at the 3-month follow-up), but there was some suggestion that teachers’ and TAs’ identification became slightly more accurate in comparison with SDQ scores. Crucially, for those pupils identified as having mental health difficulties or increased risk, in-school mental health support outcomes (ie, documentation or discussion of concerns, conversations with pupils and parents, and in-class and in-school support) increased after the training, but more “downstream” outcomes (ie, documented SEMH status and referral and access to external mental health services) did not. Qualitative findings indicated that participants generally found the training acceptable and practical, with many explaining how they intended to use or had already used the skills they learned to improve their practice. Participants also suggested several useful improvements for the training and its implementation, including making it more relevant to the United Kingdom, adding more scenarios, and including a live element in the implementation of the training.

Findings regarding confidence and preparedness reflect those of the 3 US-based studies of *At-Risk* [17,41,42] and the wider literature surrounding teacher mental health training [31]. In general, mental health training seems to be effective in improving staff confidence. For example, 2 Australian-based studies [37,78] found that secondary school teachers who completed training felt more confident discussing their concerns and helping pupils with their mental health. Another UK-based study of a psychoeducational training program to improve recognition of depression in secondary schools [79] found significant pretest-posttest improvements in teacher confidence in their knowledge of symptoms, ability to recognize symptoms, and knowledge about how to speak with pupils about their mental health. However, not all studies have shown an impact, with a prominent UK-based study of mental health first aid training finding no effect on educators’ confidence in helping pupils with their mental health [80].

The general decrease in the proportion of pupils identified as having mental health difficulties or increased risk stands in contrast to previous studies of *At-Risk*, which found that school staff identified significantly more pupils of concern after completing the training [17,41]. Evidence of the effect of other training programs on identification is extremely limited [30,31,36], and differences in context, training content and delivery, baseline knowledge, and outcome measurement make it difficult to compare findings across studies. Two vignette-based studies showed little effect of either mental health first aid [78] or psychoeducational [81] training on identification (although each study also reported high recognition of difficulties before the training), whereas studies focused on real-world identification have shown mixed results [79,82]. However, changes in the proportion of identified pupils must be contextualized within the accuracy of identification. There are consequences of both over- and underidentification [83,84], most notably in terms of inefficient allocation of limited mental health support resources. While comparison with the SDQ suggested that there was some improvement in terms of the accuracy of identification following the training, underidentification remained a substantial challenge, with between one-half and two-thirds of pupils with elevated SDQ scores remaining unidentified by teachers and TAs. The underidentification of children with mental health difficulties in educational settings, particularly for children with internalizing as opposed to externalizing problems [85], has been well documented in the literature [30], and it is likely that a combination of identification models is required to address this challenge [27,29].

Promisingly, the training appeared to be useful in terms of connecting pupils with care and support, an outcome not frequently measured in other studies [30,31,34]. First, the findings suggested that participants had conversations about or documented concerns for a greater proportion of identified pupils following the training, which reflects findings from previous studies of *At-Risk* [17,41]. This is a rather unique outcome in the literature as other training evaluations have found no difference between training and control groups in terms of conversations with pupils and colleagues [78]. Importantly, this study went beyond conversations to include outcomes pertaining to in-school and external support. The increases in in-class and in-school support for identified pupils reflect findings of the UK-based study by Kidger et al [80] of mental health first aid training and the Australian-based pilot study by Parker et al [37] of a web-based training program, each of which found a positive effect of the training on helping behaviors. Although in-class and in-school support seemed to increase following the training, it is notable that referrals and access to specialist services did not. There are several plausible explanations for this finding. For example, it is likely that school staff were already aware of children with the most severe mental health difficulties and were confident and able to support newly identified pupils—who might have had lower-level mental health difficulties—within the school setting. However, if the training *did* lead to the identification of children who might benefit from specialist care, there are many barriers to accessing such support (eg, availability and long waiting lists) that might have influenced these outcomes, as reflected in both the qualitative interviews and the wider literature [23,86].

In addition, quantitative and qualitative findings suggested that the program was a good fit for individuals and schools, which aligns with previous research on the acceptability and perceived need for mental health training for school staff [18,20,27-29,87,88]. The training’s format seemed to be a key contributor to its feasibility. With a few exceptions [37,39,89], the web-based simulation-based format of *At-Risk* is unique among training programs and is well aligned with teachers’ preferences. For example, in their focus group study of UK secondary school teachers, Shelemy et al [20] found that participants wanted engaging, interactive, and concise training that included practical strategies and illustrative case studies, all of which are central to *At-Risk*. While the authors found that teachers disagreed over the usefulness of web-based training, it is possible that these concerns would have decreased during the COVID-19 pandemic as staff became more accepting of web-based opportunities to learn.

Qualitative findings also demonstrated the importance of school context and culture, which have been highlighted in previous research [27]. In particular, participants noted the importance of school culture in adopting mental health interventions into regular practice. In their systematic review, Moore et al [90] identified school culture, values, and policies as key facilitators of sustaining mental health interventions. A related area of focus was support from the SLT. This support is a well-recognized factor contributing to intervention success and sustainability for several reasons, including these leaders’ practical role in communicating about interventions and allocating specific time and resources to them [43,90,91]. However, it is important to recognize that mental health training for school staff may be even more needed and impactful in schools where mental health is not as much of a priority.

### Limitations

Our mixed methods approach, wide range of outcomes, and diverse sample of participating schools offer rich information regarding the feasibility of *At-Risk* in the United Kingdom. These strengths notwithstanding, there are also several limitations to consider when interpreting the findings. The nonrandomized design, while common for feasibility studies, prevents any conclusions regarding causality and also limits the exploration of other factors that may have influenced outcomes (eg, providing teachers and TAs with the Mental Health Resource Maps or SENCos and mental health leads with feedback on identified pupils). In terms of recruitment and retention, the study had 50% (54/108) attrition. Several factors may have influenced this, including the increased pressure on school staff due to the COVID-19 pandemic, the timing of the study within the school year, and the requirement to communicate with participants only via the study link person. While we tried to explore the effect of attrition through a complete case sensitivity analysis, we lacked important information on the characteristics of those who dropped out as this information was collected only at T2. Furthermore, we were only able to recruit 8 staff members for the posttraining interviews, which was far below our recruitment target. Low participation rates could again be due to several factors, including the impact of the COVID-19 pandemic or competing priorities. Of note, we were not able to recruit anyone who did not complete the training, any headteachers, or any staff from 2 of the schools (schools A and D). This could mean that we lack viewpoints that may be important for understanding the feasibility and utility of the training.

There were also limitations associated with the study measures. While the Teacher and TA Identification Form was informed by the literature and reviewed by our primary school staff advisory group, its validity and reliability are unknown. In addition, the questionnaire only measured mental health support outcomes for those pupils *identified as having mental health difficulties or increased risk.* Therefore, we do not have information on those who were not identified. The measure is also based on teacher and TA reports and so may not have complete information about all types of support that pupils receive. Another important limitation pertains to the comparator used to assess the identification outcomes. To understand the potential utility of the training program, it is important to have a robust comparator. While we chose to use the teacher-report SDQ, it would also have been interesting to compare identification outcomes with parent-rated mental health difficulties, particularly in light of the low interrater agreement of common measures of child mental health difficulties [92]. An even stronger comparator would be to assess the teacher and TA identification outcomes against a clinical interview; however, this was not feasible in this study.

Finally, at the time of writing, *At-Risk* is currently not available for use as Kognito restructures its offerings. This demonstrates a trend that unfortunately is a common occurrence in the field of mental health, whereby many evidence-based digital tools are not available to potential end users [93]. Nonetheless, the learnings from this feasibility study offer rich information on what type of content and format may be useful for training programs in this area and, as such, can support further development and evaluation in the field.

### Implications for Practice

Studies have consistently demonstrated that school staff would appreciate additional training on how best to support pupil mental health [18,20,87,88]. However, to be scalable, such programs must be realistic in terms of time, cost, and resource requirements [28,90,91]. Contextualized within the wider literature on school-based mental health interventions, the findings from this study suggest that mental health training is a feasible option for upskilling school staff to identify and respond to pupil mental health difficulties. They further highlight several specific factors that might positively contribute to feasibility and scalability, many of which are reflected in the broader literature on mental health training [20,28]. For example, teachers and TAs appreciated that the training actively engaged them in learning and applying new skills and that it used realistic examples to demonstrate the real-world applicability of the training, whereas school leaders identified the relatively low time and cost requirements and flexibility as key factors that could make the training feasible for their school context.

However, this is not to say that there are no implementation barriers associated with *At-Risk* or similar training programs. While the resources required to implement *At-Risk* are relatively low compared with other training programs, they must still be considered within the context of other school priorities. As demonstrated in the interviews and the wider literature [3,43,90,91], support from school leadership is essential for securing the time and budget required to implement a training such as *At-Risk*, and in schools where mental health is not a priority, there are likely to be many barriers to implementation*.* Even in schools with strong support from the leadership team, it may be difficult to find the requisite budget, time, and human resources to devote to the training. Finally, as is the case with any school-based mental health intervention, it is important that schools do not take sole responsibility for pupils’ mental health. Active partnership between schools and mental health services is key to ensuring that schools feel empowered and supported in this role [21,90,94]. While the schools in this study worked hard to support pupils as best they could, interviewees expressed frustration about the difficulty of accessing external support for children who could benefit from it. This is not an uncommon theme in the wider literature surrounding school-based interventions [20,23,91] and is a key consideration for scaling up training programs.

### Implications for Future Research

The promising findings of this study suggest that additional research is needed to explore the role of scalable mental health training in supporting schools to protect and promote children’s mental health. On the basis of gaps in the literature, particular areas of interest include training for primary school staff (as most are focused on secondary school staff), web-based training (as opposed to traditional time- and resource-intensive in-person training), and training that takes a “whole school approach” by including all school staff members (rather than only teachers). This final area is especially interesting as findings from this study and others [27] have highlighted stakeholders’ preference that training programs include all school staff members. While our study jointly analyzed findings for teachers and TAs, future research would do well to consider how the unique roles and perspectives of these professionals—as well as other staff members within the school setting—might influence outcomes. Furthermore, future research should be more inclusive about their choice of outcomes, as too often evaluations of school staff training programs have focused on intermediate outcomes such as knowledge or confidence [31] without considering more “downstream” outcomes such as access to support. Finally, as demonstrated in our study, there is great value in using mixed methods approaches and including information about wider issues of feasibility and implementation, and studies that take this broader lens can help identify programs that are scalable, sustainable, and effective.

### Conclusions

School staff would welcome additional mental health training to enable them to respond to pupil mental health difficulties, but there are many barriers to implementing such training at scale. Therefore, training programs that have relatively low time and resource requirements have great potential to fulfill an unmet need in schools. This mixed methods feasibility study showed that *At-Risk for Elementary School Educators*—an example of a brief, interactive web-based training program—is a feasible means of empowering school staff to accurately identify and respond to pupil mental health difficulties and increased risk.

## Appendix

Multimedia Appendix 1 Teacher and Teaching Assistant Identification Form.

Multimedia Appendix 2 Kognito pre- and posttraining surveys.

Multimedia Appendix 3 Interview topic guides.

Multimedia Appendix 4 School characteristics.

Multimedia Appendix 5 Results from complete case sensitivity analysis.

Multimedia Appendix 6 Results from the sensitivity analysis excluding school D.

Multimedia Appendix 7 Quantitative and qualitative findings pertaining to the potential harms of At-Risk.

## Abbreviations

SDQ: Strengths and Difficulties Questionnaire
SEMH: social, emotional, and mental health
SENCo: special educational needs coordinator
SLT: senior leadership team
TA: teaching assistant

## Data Availability

The data sets generated during and analyzed during this study are not publicly available due to restrictions associated with our ethics approvals but are available from the corresponding author on reasonable request.
