# Supplementary material for: Empowering School Staff to Support Pupil Mental Health Through a Brief, Interactive Web-Based Training Program: Mixed Methods Study
Source: J Med Internet Res. 2024 Apr 23;26:e46764. doi: 10.2196/46764 (PMC11077415; doi:10.2196/46764)
Supplement: Multimedia Appendix 1 [file jmir_v26i1e46764_app1.pdf]

Appendix 1. Teacher/TA Identification Form

Note. Hosted on the Qualtrics online survey platform.

UNIVERSITY OF CAMBRIDGE

At-Risk Training Feasibility Study: Teacher Identification Form

We are interested in understanding who in your class **you think** might have mental health difficulties or be at risk for mental health difficulties.

What do we mean by 'mental health difficulties'?

We are broadly interested in behavioural and social-emotional problems. These include full-blown mental disorders (with a diagnosis), but can include <u>lower level problems</u> as well.

For example, a child who is very aggressive and fights a lot with other children could be considered as having behavioural difficulties, even if he/she does not have a diagnosis such as *conduct disorder*. Similarly, a child who often seems withdrawn during class and does not have any friends could qualify as having emotional difficulties, even if he/she does not have a diagnosis such as *depression*.

What do we mean by 'risk for mental health difficulties'?

There are many different things that increase a child's <u>risk</u> of developing mental health difficulties later on. These are often negative experiences in a child's life and can happen at home or at school. Examples include:

- Abuse or neglect
- Difficult home life (e.g. parents fight a lot, having a parent in prison)
- Negative life events (e.g. death of a loved one)
- Bullying

<u>Instructions</u>

On the next page, we've included a list of all the pupils in your class.

Question 1: for each pupil, the first question is about whether or not you think the pupil has mental health difficulties <u>or</u> is at risk of developing mental health difficulties. You can begin by writing Y for 'yes' or N for 'no'.

If 'no', you can move onto the next pupil. If 'yes', please answer the remaining questions.

Questions 2-6 are about what you've done about your concerns (e.g. talked to someone about them, formally documented them with the school, provided in-class support for the child). **Questions 7-10** are about whether the child receives any form of care and support, namely whether the child has (1) in-house support or a support plan, (2) documented SEMH (social, emotional and mental health needs) status, (3) a referral to specialist mental health services, or (4) current use of specialist mental health services.

For Questions 2-10, you can write **Y, N,** *or* **?**

Y = *'yes'* **N** = *'no.* **?** = *'don't know'*

We know that teachers and schools do the best they can to support their pupils, so don't worry if the answer to these questions is 'no' even if you think a child has mental health difficulties. Please try to fill out this form as honestly as possible!

Version 5: 30/08/2019

Example of how to fill out this form:

	1. Do you believe this child has mental health difficulties or risk for mental health difficulties?	2. Have you **formally documented** your concerns about the child with the school?	3. Have you spoken about your concerns with the **SENCo/pastoral care lead/mental health lead?**	4. Have you spoken about your concerns with **another member of school staff?**	5. Have you spoken about your concerns with **the child and/or his/her parents?**	6. Are you providing any special **in-class support** for this child?	7. Does this child receive **in-school support** or have an **in-house support plan**?	8. Does this child have documented **SEMH** (social, emotional and mental health needs) status?	9. Has this child been **referred** to **specialist mental health services** (e.g. emotional wellbeing team)?	10. Does this child **have access to specialist mental health services** (e.g. emotional wellbeing team)?
**Pupil 1**	Y	N	N	Y	Y	N	N	N	?	?
**Pupil 2**	N									
**Pupil 3**	Y	Y	Y	Y	Y	Y	Y	Y	N	?
**Pupil 4**	N									

Thank you for your participation in this study!

	1. Do you believe this child has mental health difficulties or risk for mental health difficulties?	2. Have you **formally documented** your concerns about the child with the school?	3. Have you spoken about your concerns with the **SENCo/pastoral care lead/mental health lead?**	4. Have you spoken about your concerns with **another member of school staff?**	5. Have you spoken about your concerns with **the child and/or his/her parents?**	6. Are you providing any special **in-class support** for this child?	7. Does this child receive **in-school support** or have an **in-house support plan**?	8. Does this child have documented **SEMH** (social, emotional and mental health needs) status?	9. Has this child been **referred** to **specialist mental health services** (e.g. emotional wellbeing team)?	10. Does this child **have access to specialist mental health services** (e.g. emotional wellbeing team)?
**Pupil 1**										
**Pupil 2**										
**Pupil 3**										
**Pupil 4**										
**Pupil 5**										
**Pupil 6**										
**Pupil 7**										
**Pupil 8**										
**Pupil 9**										
**Pupil 10**										
**Pupil 11**										
**Pupil 12**										
**Pupil 13**										
**Pupil 14**										
**Pupil 15**										
**Pupil 16**										
**Pupil 17**										
**Pupil 18**										
**Pupil 19**										
**Pupil 20**										
**Pupil 21**										
**Pupil 22**										
**Pupil 23**										
**Pupil 24**										
**Pupil 25**										
**Pupil 26**										
**Pupil 27**										
**Pupil 28**										
**Pupil 29**										
