# Supplementary material for: Empowering School Staff to Support Pupil Mental Health Through a Brief, Interactive Web-Based Training Program: Mixed Methods Study
Source: J Med Internet Res. 2024 Apr 23;26:e46764. doi: 10.2196/46764 (PMC11077415; doi:10.2196/46764)
Supplement: Multimedia Appendix 2 [file jmir_v26i1e46764_app2.pdf]

Appendix 2. Kognito pre- and post-training surveys
Note. Hosted on the Qualtrics online survey platform.

2A. PRE-TRAINING SURVEY

Before you begin the simulation, please answer the following questions. All of your data is confidential.

1. How would you rate your preparedness to:

	Very Low	Low	Medium	High	Very High
Recognise when a pupil is exhibiting signs of psychological distress (for example, depression, anxiety).					
Have a discussion with a pupil to gather more information about the signs of psychological distress they are exhibiting.					
Motivate a parent whose child is exhibiting signs of psychological distress to seek help.					
Discuss with a parent your concern about the signs of psychological distress their child is exhibiting.					
Apply communication strategies such as reflective statements and open-ended questions in discussions with parents about their child's signs of distress.					
Help parents be informed about mental health support (in school or in the community) available to a child who is exhibiting signs of psychological distress.					

2. Please rate how much you disagree/agree with the following statements:

	Strongly Disagree	Disagree	Neither Disagree nor Agree	Agree	Strongly Agree
I feel confident in my ability to have a discussion with a pupil to gather more information about the signs of psychological distress they are exhibiting.					
I feel confident in my ability to discuss with a parent my concern about the signs of psychological distress their child is exhibiting.					
I feel confident in my ability to apply communication strategies such as reflective statements and open-ended questions in discussions with parents about their child's signs of distress.					
Most teachers and staff in my school think that a pupil receiving mental health treatment is a sign of personal weakness.					
If I went to a mental health professional, I would be less satisfied with myself.					

3. In the past two academic months, approximately how many pupils have you...

been concerned about due to the signs of psychological distress they are exhibiting? ☐

approached to have a discussion to gather more information about the signs of psychological distress they are exhibiting? ☐

4. In the past two academic months, approximately how many parents have you...

talked to regarding concern about the signs of psychological distress their child is exhibiting.

had discussions with to motivate them to connect their child with mental health support services?

helped inform about mental health support (in school or in the community) available to a child who is exhibiting signs of psychological distress?

2B. POST-TRAINING SURVEY

Now that you have completed the simulation, please answer the following questions. All of your data is confidential.

1. How would you rate your <u>preparedness</u> to:

	Very Low	Low	Medium	High	Very High
Recognise when a pupil is exhibiting signs of psychological distress (for example, depression, anxiety).					
Have a discussion with a pupil to gather more information about the signs of psychological distress they are exhibiting.					
Motivate a parent whose child is exhibiting signs of psychological distress to seek help.					
Discuss with a parent your concern about the signs of psychological distress their child is exhibiting.					
Apply communication strategies such as reflective statements and open-ended questions in discussions with parents about their child's signs of distress.					
Help parents be informed about mental health support (in school or in the community) available to a child who is exhibiting signs of psychological distress.					

2. Please rate how much you disagree/agree with the following statements:

	Strongly Disagree	Disagree	Neither Disagree nor Agree	Agree	Strongly Agree
I feel confident in my ability to have a discussion with a pupil to gather more information about the signs of psychological distress they are exhibiting.					
I feel confident in my ability to discuss with a parent my concern about the signs of psychological distress their child is exhibiting.					
I feel confident in my ability to apply communication strategies such as reflective statements and open-ended questions in discussions with parents about their child's signs of distress.					
All teachers and staff in my school should take this simulation.					
Most teachers and staff in my school think that a pupil receiving mental health treatment is a sign of personal weakness.					
If I went to a mental health professional, I would be less satisfied with myself.					

3. Please indicate how much you disagree/agree with the following statement:

	Strongly Disagree	Disagree	Neither Disagree nor Agree	Agree	Strongly Agree
Applying what I learned in this simulation is likely to have a positive impact on rapport between myself and my pupils.					

4. Please indicate how much you disagree/agree with the following statements that begin with:

As a result of applying the skills learned in the simulation:

	Strongly Disagree	Disagree	Neither Disagree nor Agree	Agree	Strongly Agree
Pupil attendance will increase.					
Pupil academic success will improve.					
The learning environment/school will become safer and more supportive.					
Classroom safety will improve.					

5. Overall, how would you rate the simulation?

Poor/Good/Fair/Very good

6. Would you recommend this simulation to other educators and school staff?

Yes/No

7. Is the simulation based on scenarios relevant to you as an educator or school staff member?

Yes/No

8. What is your main employment status? (Please tick all that apply)

- Teacher
- Teaching assistant (including HLTAs)
- Member of senior leadership team
- SENCo/mental health champion/mental health lead
- School counsellor/psychologist
- School support/admin team member
- Other (please specify): ____________________

9. What is your gender?

- Male
- Female
- Non-binary or third gender
- I prefer not to answer
- Prefer to self-describe: ____________________

10. What is your age? [ ]

11. How many years have you worked in education in total? [ ]

12. How would you describe your ethnic group?

- Asian/Asian British - Any other Asian background (please describe below)
- Asian/Asian British - Bangladeshi
- Asian/Asian British - Chinese
- Asian/Asian British - Indian
- Asian/Asian British - Pakistani
- Black/African/Caribbean/Black British - African
- Black/African/Caribbean/Black British - Any other Black/African/Caribbean/Black British background (please describe below)
- Black/African/Caribbean/Black British - Caribbean
- Mixed/Multiple ethnic groups - Any other Mixed/Multiple ethnic background (please describe below)
- Mixed/Multiple ethnic groups - White and Black African
- Mixed/Multiple ethnic groups - White and Black Caribbean
- Mixed/Multiple ethnic groups -White and Asian
- Other ethnic group - any other ethnic group (please describe): ______________________
- Other ethnic group - Arab
- Prefer not to say
- White - Any other White background (please describe below)
- White - English/Welsh/Scottish/Northern Irish/British

- White - Gypsy or Irish Traveller
- White - Irish

*13. What did you like best about the simulation?

14. What would you change to make it more effective?

15. Now that you have completed the simulation, please describe a situation that you would have managed differently. What happened and what would you have done differently? Please be sure not to include any identifiable information.
