# Supplementary material for: Empowering School Staff to Support Pupil Mental Health Through a Brief, Interactive Web-Based Training Program: Mixed Methods Study
Source: J Med Internet Res. 2024 Apr 23;26:e46764. doi: 10.2196/46764 (PMC11077415; doi:10.2196/46764)
Supplement: Multimedia Appendix 3 [file jmir_v26i1e46764_app3.pdf]

Appendix 3. Interview topic guides

3A. PRE-STUDY INTERVEIW WITH SENCO/MENTAL HEALTH LEAD

Key questions to be addressed

1. What is the school environment like in terms of mental health?
2. What are the current processes and pathways (formal and informal) in place for when a staff member identifies possible mental health difficulties (MHD) in a pupil?
3. What kinds of interventions are available for children with MHD or risk for MHD in the school and in the community?
4. How will the school prepare for the possibility of increased identification following *At-Risk* training?

Key question to be addressed	Interview question
	1. What is your role at the school?<br>• How long have you worked here and what are your main responsibilities?<br>• What is your background in terms of mental health?
1	2. Is mental health generally a priority for your school?<br>• Do you believe the staff in your school generally take pupils' mental health seriously?<br>• How would you describe the 'landscape' of mental health in your pupils? For example, are there many pupils with MHD? Are certain kinds of MHD/risks for MHD more commonly identified?
2, 3	3. Does your school have formal protocols in place detailing what a school staff member should do if they think a pupil might have MHD or risk for MHD?<br>• Would you say most school staff have access to and understand these protocols (if they exist)? Are these protocols generally easy to follow?<br>• How often do pupils get referred via these formal routes (if they exist)?<br>4. Besides the formal procedures, how else do school staff members respond if they notice a pupil may have MHD or risk for MHD?<br>• Do staff members often talk to each other about these issues? Do they speak informally with you? How about with the child or his/her parents?

	5. Does your school have a list of resources teachers/TAs could use to link a pupil with support? Could we look at that list together (if it exists)?<br>• Are there any resources you feel are missing from this list that could potentially be added?
3	6. What kinds of support are available for pupils with identified MHD or risk for MHD?<br>• What kinds of programmes/interventions are available *in-school?*<br>• What kinds of support are available *outside of school*?<br>• How commonly used are these supports amongst your pupils?
4	7. After the *At-Risk* training, it's possible that teachers and TAs will notice MHD they've not picked up on before. How will the school prepare for this and what can we do, as researchers, to help?<br>• How will you ensure that teachers/TAs are aware of how to respond to potential MHD?<br>• Will there be any additional assessment of pupils who teachers/TAs identify as potentially having MHD to guard against 'false positive' identification?<br>• What resources will be available for pupils with suspected MHD?

3B. POST-STUDY INTERVIEW WITH TEACHERS AND TAS WHO COMPLETED *AT-RISK*

Key questions to be addressed

1. Is *At-Risk* training helpful for identifying and responding to MHD in children?
2. Is *At-Risk* training acceptable?
3. Is *At-Risk* training practical?
4. How does *At-Risk* training compare with current practice?
5. Are there any perceived harms associated with *At-Risk* training?

Key question to be addressed	Interview question
	1. What is your role at the school?<br>• How long have you worked here and what are your main responsibilities?<br>• Do you have any specific training in mental health? What does the training consist of?<br>• In general, would you say you're confident in dealing with mental health in the classroom?<br>• Do you think schools should be involved in the mental health of their pupils? Why or why not?
1, 2, 3, 4, 5	2. What were your overall impressions of the *At-Risk* training?<br>• Did you find *At-Risk* training helpful for identifying and responding to mental health difficulties (MHD)?<br>• How has *At-Risk* training impacted your ability to identify and respond to MHD in your pupils?<br>• Have you noticed any new MHD in your pupils as a result of the training, or had any conversations with pupils or parents about mental health? If so, how did they go?<br>• Did the training give you more confidence in identifying MHD and having conversations with pupils/parents?<br>• Overall, do you feel that the programme was beneficial for pupils?<br>3. It's also important for us to understand what happens once you've identified a pupil as having MHD/risk for MHD. Before completing the *At-Risk* training, how did you go about identifying and dealing with MHD?<br>• Does your school have clear pathways for when a staff member thinks a pupil may have MHD? Are you confident about using these pathways?<br>• If you identified any pupil(s) as having MHD/risk for MHD after completing the training, what happened next? Was there any action taken on behalf of the pupil(s)?<br>4. Did *At-Risk* add anything to your school's current practices that you described before?

2, 5	5. Overall, was *At-Risk* training a good fit for you and your school?<br>• How difficult did you find the training? Were there any parts of it that were confusing or not clear?<br>• What did you think about the format of the training (i.e. online, simulation-based)? What were the pros and cons to this format?<br>• Was the content of the training appropriate for primary school children? Is there anything you would change?<br>• How well did *At-Risk* training align with your school's priorities? Do you think the school would be willing to adopt it into regular practice? Why or why not?<br>6. Could you foresee any negatives or harms that could come about because of the training?
3	7. On the whole, was *At-Risk* training practical for you and your school, for example, in terms of resources and time requirements?<br>• What did you think of the time requirements? Was the training too long? Too short? About right? Was it a good use of your time?<br>• Did you have all of the necessary resources to complete the training? If not, what were you missing?
1, 2, 3	Now, speaking more generally and not just about your school:<br>8. Would you recommend the training to other teachers/TAs?<br>9. Going forward, do you think there's any merit in continuing to refine *At-Risk* training and use it in schools?<br>• Are there any changes you'd make to the programme that you haven't already mentioned?<br>• Are there any particular barriers you can think of in your school for the use of these types of training programmes that you haven't already mentioned above?

3C. POST-STUDY INTERVIEW WITH TEACHERS AND TAS WHO DID NOT COMPLETE *AT-RISK*

Key questions to be addressed

1. What should the role of schools be in terms of mental health?
2. How do you generally identify and respond to MHD in your pupils?
3. What were the reasons for not completing the training?
4. What were key barriers to practicality/acceptability?
5. Could the programme be adapted to fit your/your school's needs?

Key question to be addressed	Interview question
	1. What is your role at the school?<br>• How long have you worked here and what are your main responsibilities?<br>• Do you have any specific training in mental health? What does the training consist of?<br>• In general, would you say you're confident in dealing with mental health in the classroom?<br>• Do you think schools should be involved in the mental health of their pupils? Why or why not?
1	2. Thinking generally, do you think schools should be involved in the mental health of their pupils? Why or why not?<br>3. Would you say that, generally, mental health is a priority for your school?<br>• Would you say your school provides sufficient support for pupils experiencing MHD? What about for teachers who have to respond to these MHD?
1, 2	4. Does your school have clear pathways for when a staff member thinks a pupil may have MHD? If so, what are they?<br>• Are you confident about using these pathways? If not, why not?<br>5. How do you generally go about identifying and responding to MHD?<br>• What are some of the signs you tend to notice that may indicate a pupil is experiencing MHD?<br>• Who do you talk to (if anyone) when you think one of your pupils may be experiencing MHD? How did those conversations go?<br>• What actions are taken for these pupils?
3, 4	6. Can you tell me a little bit about why you didn't complete *At-Risk* training when it was offered in your school?<br>• Was there something about the programme itself (e.g. the content or format) that dissuaded you from doing the training?

	• Was there something about the school environment that kept you from completing the training? For example, were there competing priorities that took precedence over completing the training? Was there sufficient support from the school leadership team for completing the training?<br>• Are there any other major barriers you saw to completing the training?
5	7. Are there any changes you'd make to the programme that would make it more feasible or acceptable in your school, or is it 'fatally flawed'?

3D. POST-STUDY INTERVIEW WITH STRATEGIC STAKEHOLDERS

Key questions to be addressed

1. Is *At-Risk* training acceptable?
2. Is *At-Risk* training practical?
3. Are there any perceived harms associated with *At-Risk* training?
4. How does *At-Risk* training compare with current practice?

Key question to be addressed	Interview question
	1. What is your role at the school?<br>• How long have you worked here and what are your main responsibilities?<br>• What role do you play in terms of pupils' mental health?<br>• Do you have any specific training in mental health? What does the training consist of?
1, 4	2. Do you think schools should be involved in the mental health of their pupils? Why or why not?<br>3. In general, how well do you think your school looks after pupils' mental health and deals with any issues?<br>• How important is mental health in your school? Is it a priority?<br>• Does your school offer teachers/TAs any training about mental health?<br>• Does your school have any programmes that promote positive mental health?
1, 4	4. We know that, in general, most MHD in children go unidentified (for a number of different reasons). How does your school currently go about identifying pupils with MHD?<br>• Does your school have clear pathways for when a staff member thinks a pupil may have MHD? If so, what are they? How often are they utilised by teachers or TAs?<br>• Are schools well-placed to identify MHD?
1, 2, 3, 4	5. What were your general impressions of the *At-Risk* training?<br>• Does *At-Risk* add anything to your school's current practices that you described before?<br>• How well did *At-Risk* training align with your school's priorities?<br>• Was *At-Risk* training appropriate for staff and pupils in your school?<br>6. Did you personally notice any differences in attitudes toward mental health, identification rates, or other aspects of mental health after the training?

	7. Could you foresee any negatives or harms that could come about because of the training?
2	8. Was *At-Risk* training practical for your school?<br>• What did you think about the online, one-off, simulation-based format of the training?<br>• *At-Risk* training takes approximately 1 hour to complete. What do you think of this time requirement? Would you consider it a good use of time for teachers and TAs?<br>• Did your school have all of the necessary resources to offer the training? If not, what were you missing?<br>• Would you be willing to pay for this programme?
1, 2, 3, 4	9. Do you think that your school would be willing to adopt *At-Risk* training into regular practice? Why or why not?<br>• How easy/difficult is it generally to adopt new programmes or initiatives into your school?<br>10. Overall, would you recommend the training to other schools?<br>11. Going forward, do you think there's any merit in continuing to refine *At-Risk* training and use it in schools?<br>• Are there any changes you'd make to the programme that you haven't already mentioned?<br>• Are there any particular barriers you can think of in your school for the use of these types of training programmes that you haven't already mentioned?
