# Supplementary material for: Empowering School Staff to Support Pupil Mental Health Through a Brief, Interactive Web-Based Training Program: Mixed Methods Study
Source: J Med Internet Res. 2024 Apr 23;26:e46764. doi: 10.2196/46764 (PMC11077415; doi:10.2196/46764)
Supplement: Multimedia Appendix 4 [file jmir_v26i1e46764_app4.pdf]

Appendix 4. School characteristics

Table 4.1. *Characteristics of schools included in the study*

	School A	School B	School C	School D	School E	School F
**Community indicator**						
County	Merseyside	Greater London	Cambridgeshire	Cambridgeshire	Cambridgeshire	Cambridgeshire
Rural vs. urban	Urban	Urban	Rural	Urban	Urban	Urban
School type	Academy sponsor led	Academy converter	Community school	Foundation school	Voluntary aided school	Academy sponsor led
Sex	Girls only	Mixed	Mixed	Mixed	Mixed	Mixed
Area IMD[a]	4	2	3	9	2	2
Ethnicity[b]						
% Asian or Asian British	3.5	8.4	1.3	4.7	20.6	8.1
% Black, Black British, Caribbean, or African	0.8	22.5	0	1.5	6.7	3.0
% Mixed or multiple ethnic groups	3.7	6.6	1.3	9.1	3.3	5.7
% White	88.0	47.2	97.4	83.2	66.0	82.2
% Other ethnic group	0.9	12.5	0	0.2	3.3	0
**School indicator**						
Funding	State funded	State funded	State funded	State funded	State funded	State funded
Pupils (rounded)	Not available	> 1100	< 100	~400	~200	~300
% eligible for free school meals	[e]	19.8	23.7	3.5	8.1	27.7
% SEND[c]	[e]	21.9	6.6	12.8	6.2	17.5
% SEMH as primary SEN need[d]	[e]	4.7	1.3	0.5	1.4	4.0

[a] IMD=Index of Multiple Deprivation [39], whereby lower deciles represent relatively more deprived areas

[b] Ethnic categories reflect those of the 2021 census (https://www.ethnicity-facts-figures.service.gov.uk/style-guide/ethnic-groups), adjusted for available data in the 2019 school characteristics file: Asian or Asian British=Indian, Pakistani, Bangladeshi, Chinese, any other Asian background; Black, Black British, Caribbean, or African=Caribbean, African, any other Black background; Mixed or multiple ethnic groups=White and Black Caribbean, White and Black African, White and Asian, any other Mixed or multiple ethnic background; White=White British, Irish, Irish Traveller, Gypsy/Roma, any other White background; Other ethnic group=any other ethnic group. Totals <100% are due to some children's ethnicity being recorded as 'unclassified'

[c] SEND=special educational needs and disability (defined as having a learning difficulty or disability requiring special educational provision)

[d] SEMH=social, emotional, and mental health needs

[e] Data for School A not distinguishable between primary and secondary school.
