# Supplementary material for: Empowering School Staff to Support Pupil Mental Health Through a Brief, Interactive Web-Based Training Program: Mixed Methods Study
Source: J Med Internet Res. 2024 Apr 23;26:e46764. doi: 10.2196/46764 (PMC11077415; doi:10.2196/46764)
Supplement: Multimedia Appendix 5 [file jmir_v26i1e46764_app5.pdf]

Appendix 5. Results from complete cases sensitivity analysis

Complete cases sensitivity analysis

In this appendix, we present results for the complete cases sensitivity analysis, which includes all participants who completed T1, T2, and T3 questionnaires.

Teacher/TA self-efficacy and preparedness

The following three figures show findings concerning preparedness (Figure 5.1), self-efficacy (Figure 5.2), and perceptions of the impact of the training (Figure 5.3). They show that teachers and TAs reported increased preparedness and self-efficacy to identify and respond to concerns following the training and that they believed applying the skills from the training would have a positive impact on their relationships with pupils, pupil outcomes, and classroom environment.

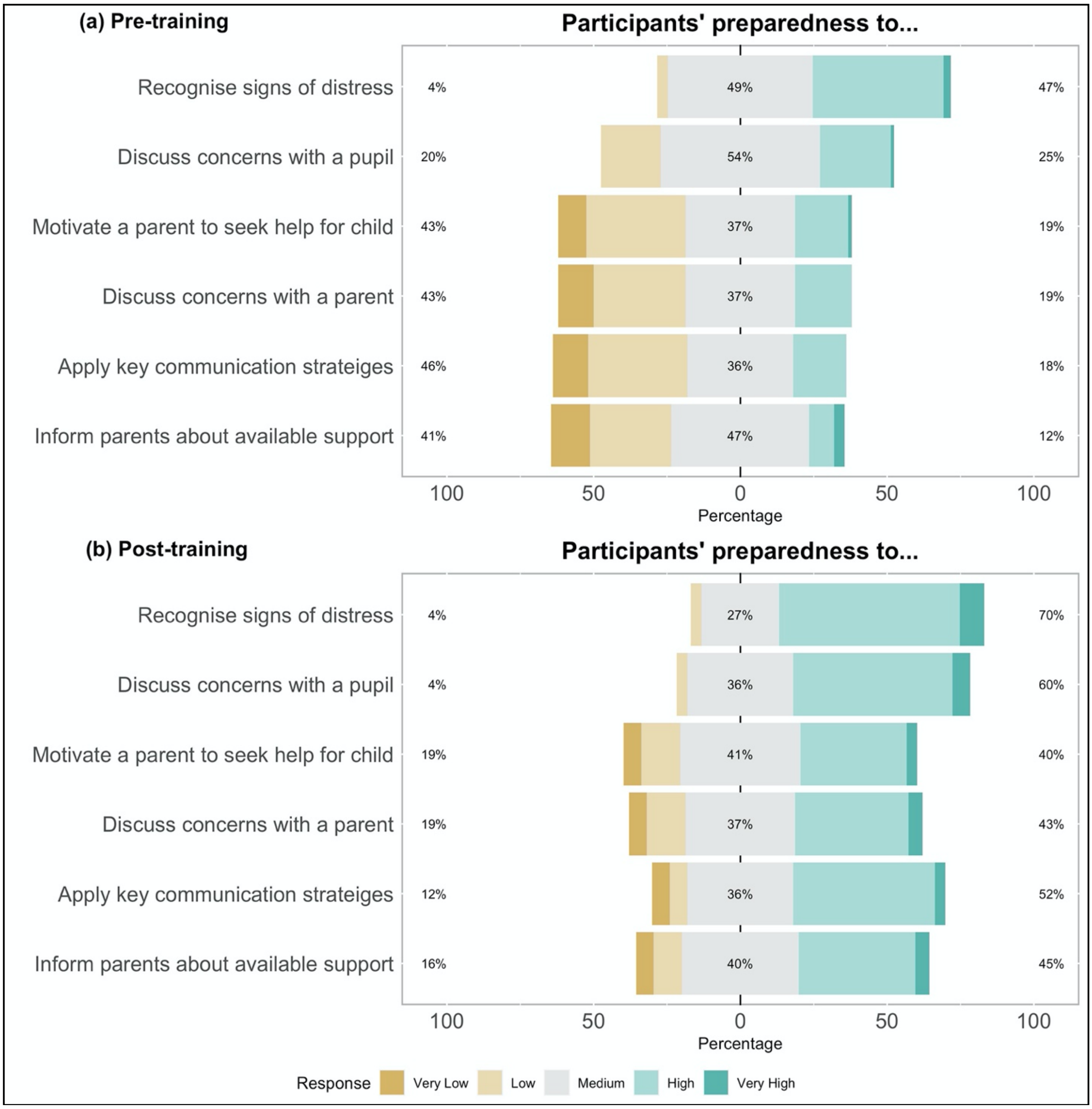

Figure 5.1. Preparedness outcomes for the complete cases sensitivity analysis (N = 83)

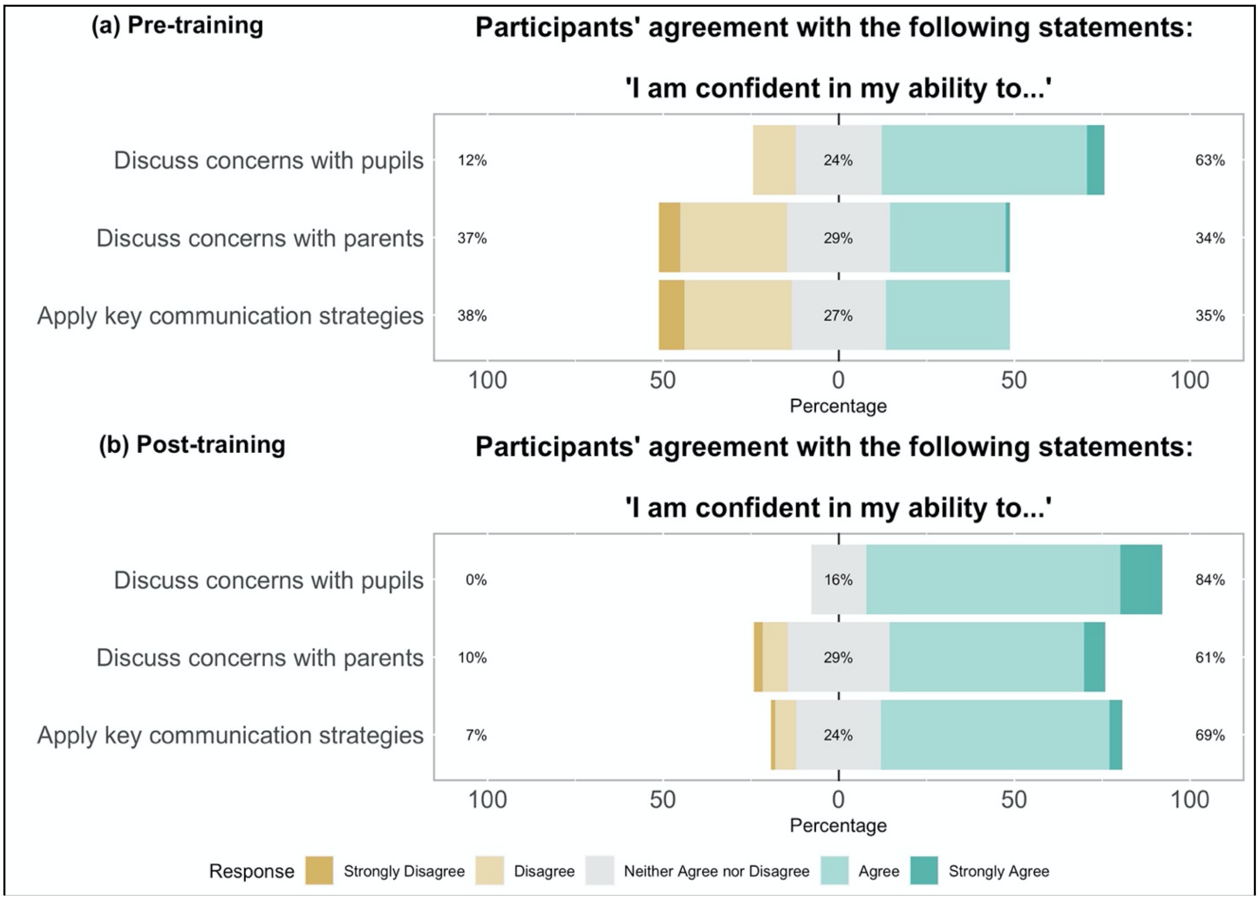

Figure 5.2. Self-efficacy outcomes for the complete cases sensitivity analysis (N = 83)

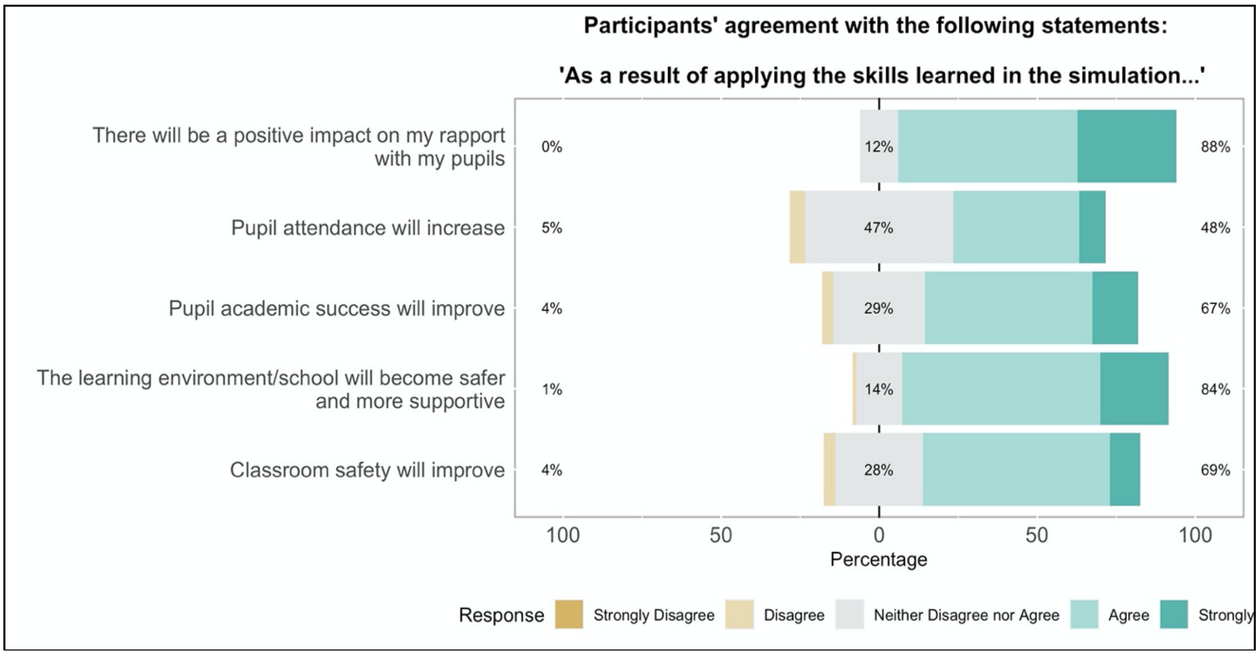

Figure 5.3. Perceptions of training impact for the complete cases sensitivity analysis (N = 83)

Identification outcomes

Table 5.1 below presents the findings for identification outcomes. The median percentage of pupils identified as having mental health difficulties or increased risk slightly decreased across the three times points. The findings comparing teacher/TA identification with SDQ scores are the same as for the main analysis (see *Analysis* section for rationale).

Table 5.1. Percentage of class identified by teachers/TAs as having mental health difficulties or risk for mental health difficulties

Outcome	**T1 (pre-training)** Median (IQR) N = 51	**T2 (1 wk. post-training)** Median (IQR) N = 51	**T3 (3 mo. post-training)** Median (IQR) N = 51
**Percentage of class identified as having mental health difficulties or increased risk**	10.0 (10.6)	8.0 (12.8)	7.4 (10.9)

Mental health support outcomes

Figure 5.4 below presents the findings for mental health support outcomes. In general, the percentage of identified children about whom teachers and TAs communicated concerns (formally or informally) increased after the training, as did the percentage receiving in-school or in-class support. Again, there was substantial variation in outcomes. The more 'downstream' support outcomes (i.e. SEMH status and referral/access to external mental health services) stayed relatively constant.

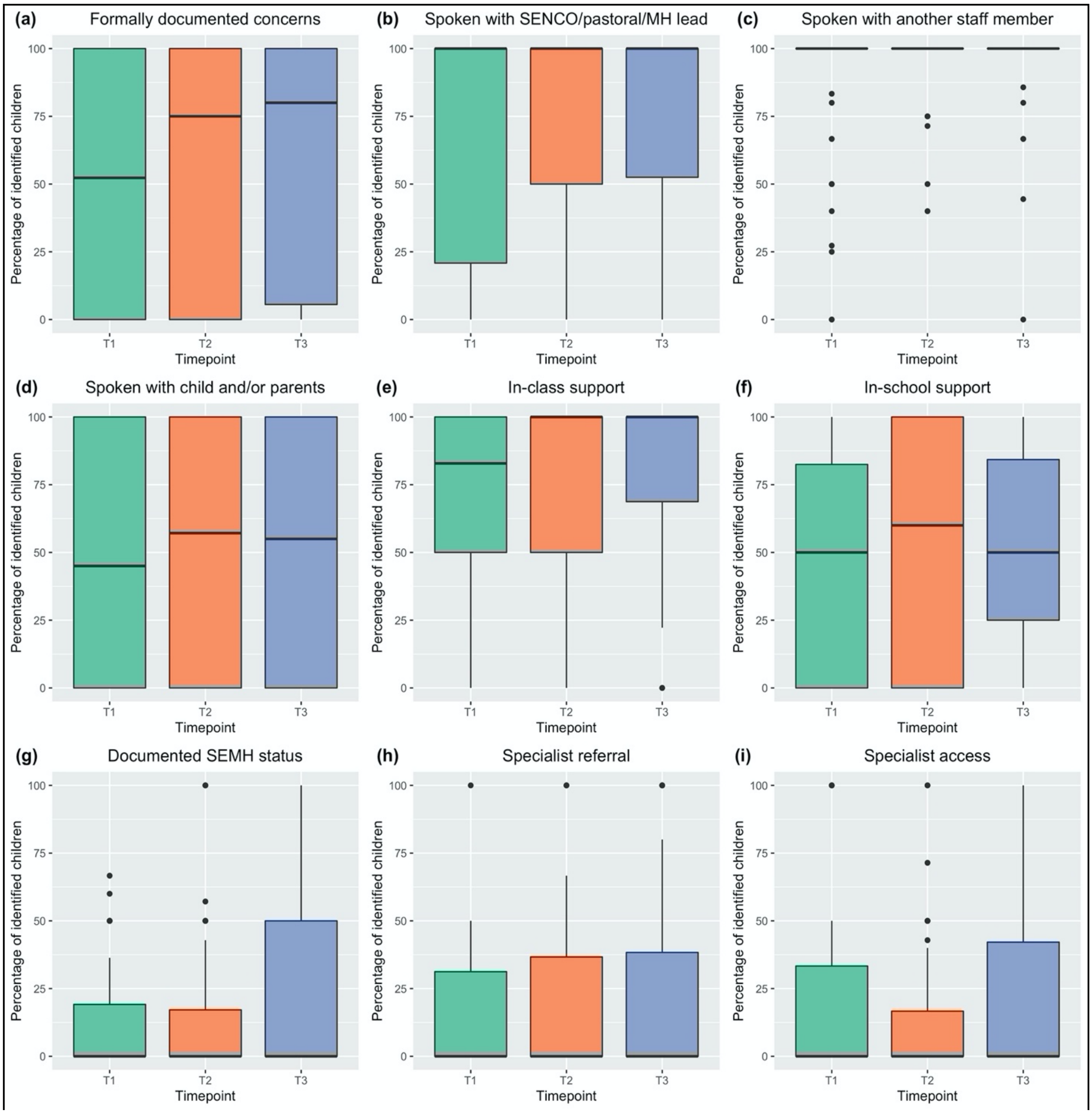

Figure 5.4. Mental health support outcomes for the complete cases sensitivity analysis
Note. Outcomes exclude those participants who were not concerned about any child (N_{T1} = 1, N_{T2} = 4, N_{T3} = 5).
