# Supplementary material for: Empowering School Staff to Support Pupil Mental Health Through a Brief, Interactive Web-Based Training Program: Mixed Methods Study
Source: J Med Internet Res. 2024 Apr 23;26:e46764. doi: 10.2196/46764 (PMC11077415; doi:10.2196/46764)
Supplement: Multimedia Appendix 6 [file jmir_v26i1e46764_app6.pdf]

Appendix 6. Results from sensitivity analysis excluding School D

Sensitivity analysis excluding School D

In this appendix, we present results for the sensitivity analysis excluding School D, which had a slightly different version of the Teacher/TA Identification Form compared with the other participating schools.

Teacher/TA self-efficacy and preparedness

These findings are not included because the difference in the Teacher/TA Identification Form would have no bearing on these results.

Identification outcomes

Table 6.1 below presents the findings for identification outcomes. The median percentage of pupils identified as having mental health difficulties or increased risk remained constant from T1 to T2 but decreased by T3.

Table 6.1. Percentage of class identified by teachers/TAs as having mental health difficulties or risk for mental health difficulties

Outcome	**T1 (pre-training)** Median (IQR)<br><br>N = 70	**T2 (1 wk. post-training)** Median (IQR)<br><br>N = 54	**T3 (3 mo. post-training)** Median (IQR)<br><br>N = 41
**Percentage of class identified as having mental health difficulties or increased risk**	12.4 (12.5)	12.3 (13.2)	8.0 (16.7)

Table 6.2 presents the findings for the comparison between teacher/TA identification and SDQ scores. The percentage of children identified by teachers and TAs who *did not* have elevated SDQ scores decreased by approximately 20 percentage points from T1 to T3. The percentage of children *with elevated* SDQ scores who *were not* identified decreased by over 10 percentage points from T1 to T3. However, for both of these outcomes, the interquartile ranges were large, indicating substantial variation across participants.

Table 6.2. Comparisons of Teacher/TA Identification Form and SDQ

Outcome	**T1 (pre-training)** Median (IQR)	**T2 (1 wk. post-training)** Median (IQR)	**T3 (3 mo. post-training)** Median (IQR)
**Percentage of children identified by teachers and TAs who do not have elevated SDQ scores** (N = 22)	40.0 (50.0)	0 (50.0)	22.5 (51.1)
**Percentage of children with elevated SDQ scores who were not identified by teachers and TAs** (N = 21)	68.8 (43.3)	62.5 (40.5)	56.3 (53.3)

Mental health support outcomes

Figure 6.1 below presents the findings for mental health support outcomes. In general, the percentage of identified children about whom teachers and TAs communicated concerns (formally or informally) increased after the training, as did the percentage receiving in-school or in-class support. Again, there was substantial variation in outcomes. The more ‘downstream’ support outcomes (i.e. SEMH status and referral/access to external mental health services) stayed relatively constant.

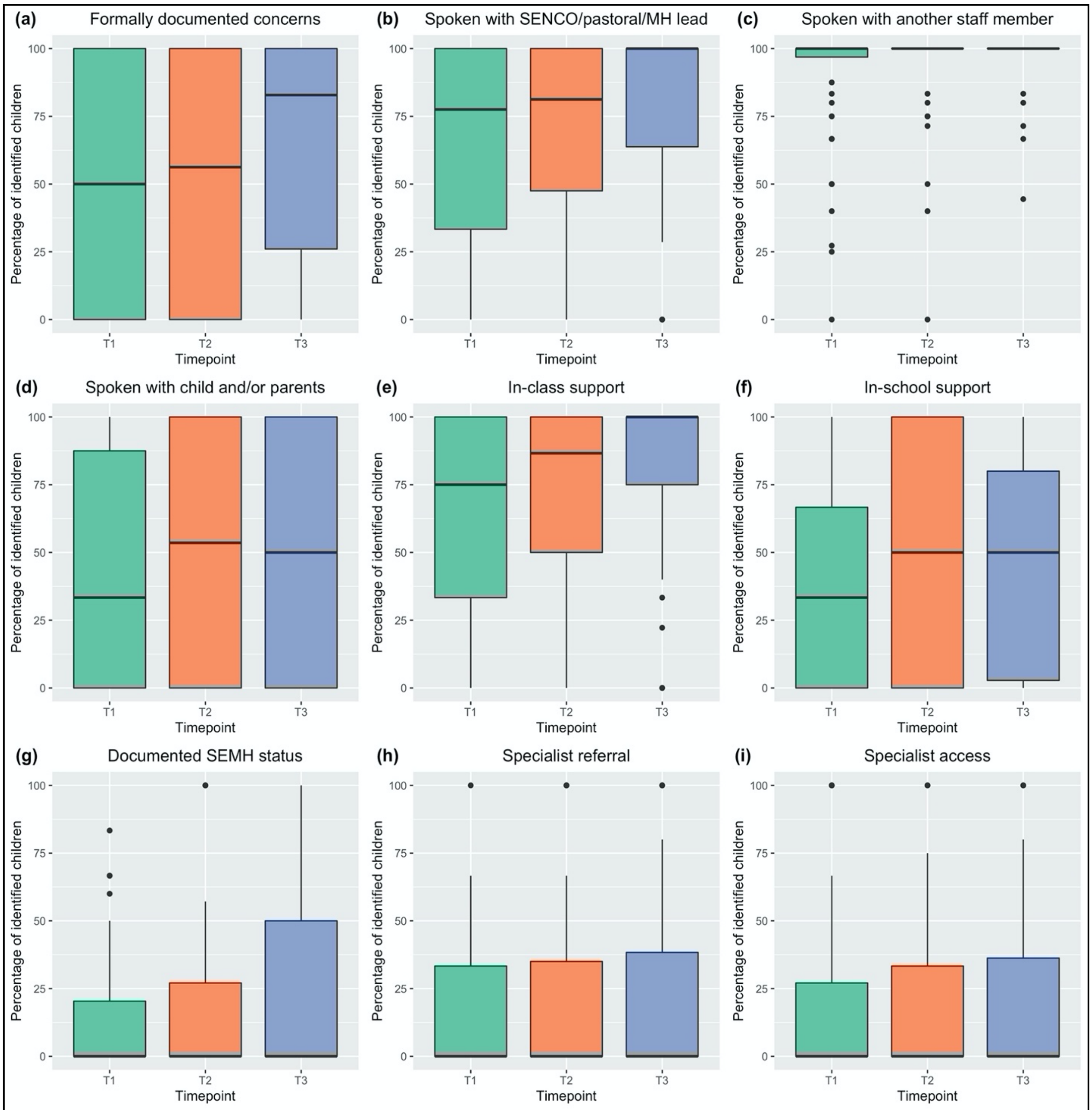

Figure 6.1. Mental health support outcomes for the sensitivity analysis excluding School D
Note. Outcomes exclude those participants who were not concerned about any child (N_{T1} = 2, N_{T2} = 2, N_{T3} = 3).
