# Supplementary material for: Empowering School Staff to Support Pupil Mental Health Through a Brief, Interactive Web-Based Training Program: Mixed Methods Study
Source: J Med Internet Res. 2024 Apr 23;26:e46764. doi: 10.2196/46764 (PMC11077415; doi:10.2196/46764)
Supplement: Multimedia Appendix 7 [file jmir_v26i1e46764_app7.pdf]

Appendix 7. Quantitative and qualitative findings pertaining to potential harms of *At-Risk*

Quantitative and qualitative findings pertaining to potential harms of *At-Risk*

The first aspect of harms pertained to rates of inaccurate identification. False positive and negative results may be associated with potential harms: the former may cause undue distress for children and families, whilst the latter might mean that pupils who could benefit from support go undetected. However, as there was some suggestion that these outcomes may have *improved* after training, this does not appear to be a specific harm associated with completing *At-Risk.*

In the qualitative interviews, participants raised few potential harms pertaining to the training, and generally viewed them as tractable problems that did not pose a high risk. In terms of harms for staff, one of the more common concerns was that the training could be triggering for those who had their own mental health difficulties. One SENCo shared that two staff members in her school chose not to take part in the training due to their own mental health. Participants suggested that content warnings and assurances that staff could discontinue the training at any time could help mitigate this harm.

In terms of harms for children, some participants worried that the training might lead school staff to misjudge situations or give them too much confidence to intervene. Two examples of this were as follows:

> My only concern was that if people could maybe jump to a conclusion. They could possibly see a behaviour that maybe they'd seen in the training, and say, 'OK so this must be the problem,' which it may well be, but it's just I think that speaking it through with someone, monitoring… of course it does need to be raised, but not just maybe putting labels on… [Interviewee 1]

> So say for instance, somebody that really didn't have the personal skills to talk with a child with potentially emotional or mental health [difficulties] […] if they hadn't have done the training, they might have signposted them [to relevant staff], but they don't do that because they've had the training and they think, 'Oh I can deal with this' and then maybe something is, something bigger is missed because of it. [Interviewee 7]

Each of these participants noted that the impact of *At-Risk* on staff knowledge and confidence may inadvertently cause them to take action that may cause them to overlook wider patterns in a pupil's behaviour or demeanour. Addressing these concerns is important for ensuring that the training does not have unintended consequences.
